# Emergent ecological patterns and modelling of gut microbiomes in health and in disease

**DOI:** 10.1371/journal.pcbi.1012482

**Published:** 2024-09-27

**Authors:** Jacopo Pasqualini, Sonia Facchin, Andrea Rinaldo, Amos Maritan, Edoardo Savarino, Samir Suweis

**Affiliations:** 1 Dipartimento di Fisica “G. Galilei” e INFN sezione di Padova, University of Padova, Padova, Italy; 2 Dipartimento di Scienze Chirurgiche, Oncologiche e Gastroenterologiche (DiSCOG), University of Padova, Padova, Italy; 3 Dipartimento di Ingegneria Civile, Edile e Ambientale (ICEA), University of Padova, Padova, Italy; 4 Laboratory of Ecohydrology, École Polytechnique Fédérale Lausanne, Lausanne, Switzerland; Emory University Department of Biology, UNITED STATES OF AMERICA

## Abstract

Recent advancements in next-generation sequencing have revolutionized our understanding of the human microbiome. Despite this progress, challenges persist in comprehending the microbiome’s influence on disease, hindered by technical complexities in species classification, abundance estimation, and data compositionality. At the same time, the existence of macroecological laws describing the variation and diversity in microbial communities irrespective of their environment has been recently proposed using 16s data and explained by a simple phenomenological model of population dynamics. We here investigate the relationship between dysbiosis, i.e. in unhealthy individuals there are deviations from the “regular” composition of the gut microbial community, and the existence of macro-ecological emergent law in microbial communities. We first quantitatively reconstruct these patterns at the species level using shotgun data, and addressing the consequences of sampling effects and statistical errors on ecological patterns. We then ask if such patterns can discriminate between healthy and unhealthy cohorts. Concomitantly, we evaluate the efficacy of different statistical generative models, which incorporate sampling and population dynamics, to describe such patterns and distinguish which are expected by chance, versus those that are potentially informative about disease states or other biological drivers. A critical aspect of our analysis is understanding the relationship between model parameters, which have clear ecological interpretations, and the state of the gut microbiome, thereby enabling the generation of synthetic compositional data that distinctively represent healthy and unhealthy individuals. Our approach, grounded in theoretical ecology and statistical physics, allows for a robust comparison of these models with empirical data, enhancing our understanding of the strengths and limitations of simple microbial models of population dynamics.

## Author summary

In this study, we explore emerging ecological properties in gut microbiomes. Our aim here is to determine whether these patterns can be informative of the gut microbiome (healthy or diseased) and unveil essential ingredients driving its population dynamics. Leveraging on metagenomic data and interpretable statistical models based on ecological processes, we show that not all ecological patterns are informative to characterize its states, while few are (e.g., species diversity). Eventually, thanks to the ecological interpretability of the inferred models’ parameters, our analysis provides insights into the role of environmental fluctuations and carrying capacities of the gut microbiomes in both health and disease. This study offers valuable knowledge, bridging theoretical concepts with practical implications for human health.

## Introduction

Next-generation sequencing has expanded our capacity to explore microbial biodiversity in a previously unachievable depth. This ‘data explosion’ presents both challenges and exciting prospects. Over the past 15 years, biomedical researchers have leveraged this technology to delve into the human microbiome—the complex ecosystem of microorganisms coexisting in and on the human body [1–4]. This approach has illuminated countless microorganisms, once inaccessible via conventional culturing methods. All these efforts have aimed to establish a community resource program to build comprehensive reference datasets and develop computational tools and clinical protocols. Although several recent studies underscore the critical role of the microbiome in human health [5–11], our understanding of how the microbiome influences disease is still limited. Current methods, primarily focused on correlations and associations within the microbiome, are useful but often fail to identify the actual causes behind these patterns [12]. In part, this is also due to several technical challenges in species classification and abundance estimations, like sampling effects, false positives species (type 1 statistical error in species detection) and data compositionality. In fact, capturing only sample fragments of the entire genetic material leads to sparse datasets, where zero abundances do not always imply species absence [13, 14]. Taxonomic profiling introduces false positive species due to genome sequence overlaps, often resolved by selecting appropriate databases or setting abundance cut-offs [15]. Additionally, normalization, such as sum-to-one, is essential due to the compositional nature of microbiome data, significantly impacting data analysis [16–18]. Nevertheless, microbiomes data display several emergent ecological patterns that are suitable to be explained through population dynamics models. Such models range from probabilistic ones, like the Multinomial Dirichlet Distribution [19], which estimates relative abundances and considers sampling effects, to more complex interaction-based models like the generalized Lotka-Volterra model or other phenomenological/computational models based on inferring species interactions [20–23]. Finally, non-interacting stochastic models reflecting basic ecological processes, like the stochastic logistic model [24], have demonstrated their effectiveness in reproducing microbiomes macroecological patterns [13].

However, an investigation of a possible link between such emergent patterns and the state space of the human gut microbiome, including dysbiosis, is still missing. This study intends to address this gap by integrating theoretical frameworks in population dynamics modelling with empirical data on gut microbiomes in health and in disease. In particular, our work aims to: 1) Quantitatively reconstruct gut microbiome emergent patterns [25] in health and in disease at the species level using shotgun data through a recently proposed taxonomic classifier [26]; In particular, our analysis includes a meta-analysis of studies on gut microbiomes in healthy individuals and those with gastrointestinal diseases [4, 10, 27]. 2) Examine the consequences of sampling effects and false positives on such ecological patterns; 3) Develop and compare how well different statistical interpretable ecological models can describe such patterns and test possible differences between healthy and diseased cohorts; 4) Understand possible relationships of the inferred models parameters (having a well-defined ecological interpretation) with gut microbiome state (e.g., health or disease), so to be able to generate synthetic compositional data with statistically significant differences between healthy and unhealthy individuals. In this way, we can distinguish which patterns are “inevitable” versus those that are potentially informative about disease states or other biological drivers.

## Materials and methods

### Theoretical framework

We now implement the mathematical framework, which can be summarized as follows. Starting from a set of equations describing the dynamics of species abundances, we develop three different statistical models aimed at generating synthetic data that match the observed data. We then will use macroecological relationships to constrain these models, leaving a small number of free parameters that can be fit to empirical data. By fitting these models and comparing their predictions against data from healthy and unhealthy microbiomes, we will eventually be able to gain insight into how different ecological patterns may arise and if and how the parameters inferred from the models are related to the state (H or U) of the gut microbiome.

We therefore introduce the stochastic logistic model [13, 24], which gives the evolution of *S* species abundances in time

$$\begin{array}{c} \frac{dx_{i}}{dt}=\frac{x_{i}}{\tau_{i}}\left(1-\frac{x_{i}}{K_{i}}\right)+\sqrt{\frac{\sigma_{i}}{\tau_{i}}}x_{i}\xi_{i}, \end{array}$$ (1)

where *x*_*i*_ ∈ (0, ∞), *K*_*i*_ is the carrying capacity of species *i* = 1…*S*, *τ*_*i*_ sets the growth time scale and *σ*_*i*_ is the width of environmental noise experienced by the *i*-th species. The latter captures the fluctuations induced to the species growth rate by the environment (e.g. host) and by species interactions [28].

It can be shown that this process, once stationarity is reached, follows a Gamma *Distribution*, i.e. $p_{\Gamma }\left(x_{i}\right)=\frac{\beta_{i}^{\alpha_{i}}}{\Gamma (\alpha_{i})}x_{i}^{\alpha_{i}-1}e^{-\beta_{i}x_{i}}$, which describes the abundance fluctuations of species *i* among different samples, without considering compositionality [16, 29] and sampling effects [14]. Generally, we will refer to the distribution of abundance of a given species across different samples as abundance fluctuation distribution [13]. The parameters *α*_*i*_ and *β*_*i*_ can be related to the statistical mean and variance of the species abundances and the ecological parameters given by the model as

$$\begin{array}{cc} \alpha_{i} & =\frac{{\bar{x}}_{i}^{2}}{\sigma_{x_{i}}^{2}}=\frac{2-\sigma_{i}}{\sigma_{i}}\;,\; \end{array}$$ (2)

$$\begin{array}{cc} \underset{\text{Parameter}}{\underset{︸}{\beta_{i}}} & =\underset{\text{Observables}}{\underset{︸}{\frac{{\bar{x}}_{i}}{\sigma_{x_{i}}^{2}}}}=\underset{\text{Ecology}}{\underset{︸}{\frac{2}{\sigma_{i}K_{i}}}} \end{array}$$ (3)

Also, we can write the first two moments of the Gamma distribution as ${\bar{x}}_{i}=K_{i}\left(1-\frac{\sigma_{i}}{2}\right)$ and $\sigma_{x_{i}}^{2}=K_{i}^{2}\frac{\sigma_{i}}{2}\left(1-\frac{\sigma_{i}}{2}\right)$. In other words, Eqs (2) and 3 show how the parameters of the Gamma distribution (*α*_*i*_,*β*_*i*_) are related to the observables (${\bar{x}}_{i}$, $\sigma_{x_{i}}^{2}$) and to the ecological parameters *K*_*i*_ and *σ*_*i*_. If we now consider compositionality and work with the relative abundances $v_{i}=x_{i}/\sum_{i=1}^{S}x_{i}$, then the latter are distributed following the Scaled Dirichlet Distribution [30]

$$\begin{array}{c} P\left(\vec{v}|\vec{\alpha },\vec{\beta }\right)=\frac{1}{Z(\vec{\alpha },\vec{\beta })}\frac{\prod_{i=1}^{S}v_{i}^{\alpha_{i}-1}}{{\left(\sum_{i=1}^{S}\beta_{i}v_{i}\right)}^{\alpha_{0}}}, \end{array}$$ (4)

where the distribution is defined with the constraint $\sum_{i=1}^{S}v_{i}=1$. We have also introduced $\alpha_{0}=\sum_{i=1}^{S}\alpha_{i}$, and the normalization constant $Z\left(\vec{\alpha },\vec{\beta }\right)=B\left(\vec{\alpha }\right)/\prod_{i=1}^{S}\beta_{i}^{\alpha_{i}}$. Finally, $B\left(\vec{\alpha }\right)=\prod_{i=1}^{S}\Gamma \left(\alpha_{i}\right)/\Gamma \left(\sum_{i=1}^{S}\alpha_{i}\right)$ is the *S*-variate Beta function. An exhaustive derivation of this result can be found in S2 Text. The obtained family of probability distributions $P(\vec{v}|\vec{\alpha },\vec{\beta })$ describes the behavior of the *relative species abundances* in a given sample, has 2*S* parameters, and lies in the (*S* − 1)-dimensional simplex *Δ*^*S*^. Since the number of observed species is large (*S* ∈ [10^2^, 10^3^]) and the microbiome dataset typically includes *R* ≈ 10^2^ samples, fitting this model is an unfeasible task. As we will show, we can greatly reduce the number of free parameters by constraining the model through relationships obtained from empirical macro-ecological patterns, as proposed by [13].

First, we consider the Taylor’s Law (TL). It takes the form of a scaling relation between the mean abundance of a species (among samples) and its fluctuations, i.e.

$$\begin{array}{c} \sigma_{x_{i}}^{2}=A{\bar{x}}_{i}^{\zeta }, \end{array}$$ (5)

for *i* = 1…*S*, where *A* and *ζ* do not depend on *i*. Compositionality does not affect this law if and only if *ζ* = 2, and thus in this case the same *A* is also found if we consider $\vec{v}$, instead of $\vec{x}$ (otherwise a correction of the slope *A* should be taken into account, see S2 Text). Therefore, we can connect the ecological parameters *K*_*i*_, *σ*_*i*_ with *ζ* = 2, finding that TL is informative on both intra-species competition (driven by *K*) and the intensity of environmental noise (*σ*).

Exploiting Eqs (2) and 3, we can thus reduce the number of parameters in our Scaled Dirichlet Distribution model to 2 + *S*, since *α*_*i*_ and *β*_*i*_ are functions of ${\bar{x}}_{i}$, *ζ* and *A*:

$$\begin{array}{c} \begin{array}{cc} \alpha_{i} & =\frac{{\bar{x}}_{i}^{2-\zeta }}{A}\;,\;\beta_{i}=\frac{{\bar{x_{i}}}^{1-\zeta }}{A}. \end{array} \end{array}$$ (6)

The dependence of the *α*s and *β*s on the exponent *ζ* suggests that there exist two interesting behaviors for the Scaled Dirichlet Distribution. In fact, for *ζ* = 1 we have a Poisson-like scaling as variance and mean are proportional, and the Scaled Dirichlet Distribution reduces to the Dirichlet distribution. The other limiting behavior with *ζ* = 2 is classically encountered in theoretical ecology [31]. In the following, we will only consider these two limiting cases, reducing the number of free parameters to 1 (the TL’s amplitude *A*) + *S* (the species mean abundances). Therefore, after some manipulations, we obtain the following distributions:

$$\begin{array}{cc} P_{\zeta =1}\left(\vec{v}|\vec{\alpha }\right) & =\frac{1}{B(\vec{\alpha })}\;\prod_{i=1}^{S}v_{i}^{\alpha_{i}-1} \end{array}$$ (7)

$$\begin{array}{cc} P_{\zeta =2}\left(\vec{v}|S,\alpha ,\vec{\beta }\right) & =\frac{1}{Z(S,\alpha ,\vec{\beta })}\;\frac{{\left(\prod_{i}\;v_{i}\right)}^{\alpha -1}}{{\left(\sum_{i}\;\beta_{i}v_{i}\right)}^{\alpha S}}\;, \end{array}$$ (8)

where ∑_*i*_ *v*_*i*_ = 1. The *ζ* = 1 prescribes a Poisson-like scaling with $\alpha_{i}=\frac{{\bar{x}}_{i}}{A}$ (${\bar{x}}_{i}$ and *A* can be directly obtained from the data), and all the *β*s are proportional to a constant, and are canceled out from the analytic expression of the Dirichlet distribution given by Eq (7). On the other hand, for *ζ* = 2 we find that all *α*_*i*_ = *α* = (2 − *σ*)/*σ* = *A*^−1^ are constant and $\beta_{i}∼K_{i}^{-1}$. Due to the invariance of the corresponding distribution by rescaling of the *β*s, the proportionality constant is irrelevant (see SM section 4.2.4). Also, because in the latter case *α*_*i*_ = *α*, then the dependence of $Z(\vec{\alpha },\vec{\beta })$ on $\vec{\alpha }$ reduces to $Z(S,\alpha ,\vec{\beta })$ and thus the corresponding joint pdf given by Eq (8) is the Symmetric Scaled Dirichlet distribution.

The second pattern that we exploit to reduce the number of the models free parameters is the species *mean* (relative) *abundance distribution* (MAD), which describes the frequencies of the species abundances averaged over samples. Indeed, it has been shown that different types of microbiomes share the same average (relative) species abundance distribution [13], i.e., ${\bar{x}}_{i}∼P_{\text{MAD}}\left(\mu ,\lambda \right)$ (${\bar{v}}_{i}∼P_{\text{MAD}}\left(\mu_{v},\lambda_{v}\right)$), where the parameters *μ* (*μ*_*v*_) and λ (λ_*v*_) can be inferred from the data. In S2 Text, we show that in the large *S* limit λ_*v*_ ≈ λ. Typically, the distribution *P*_MAD_ has fat tails, and it is compatible with a Log-Normal distribution [13]. Regardless of the particular form *P*_MAD_ takes, its presence allows us to generate all the ${\bar{x}}_{i}$ (*i* = 1, …*S*) once *μ* and λ are fitted from the data.

**Fig 1:**
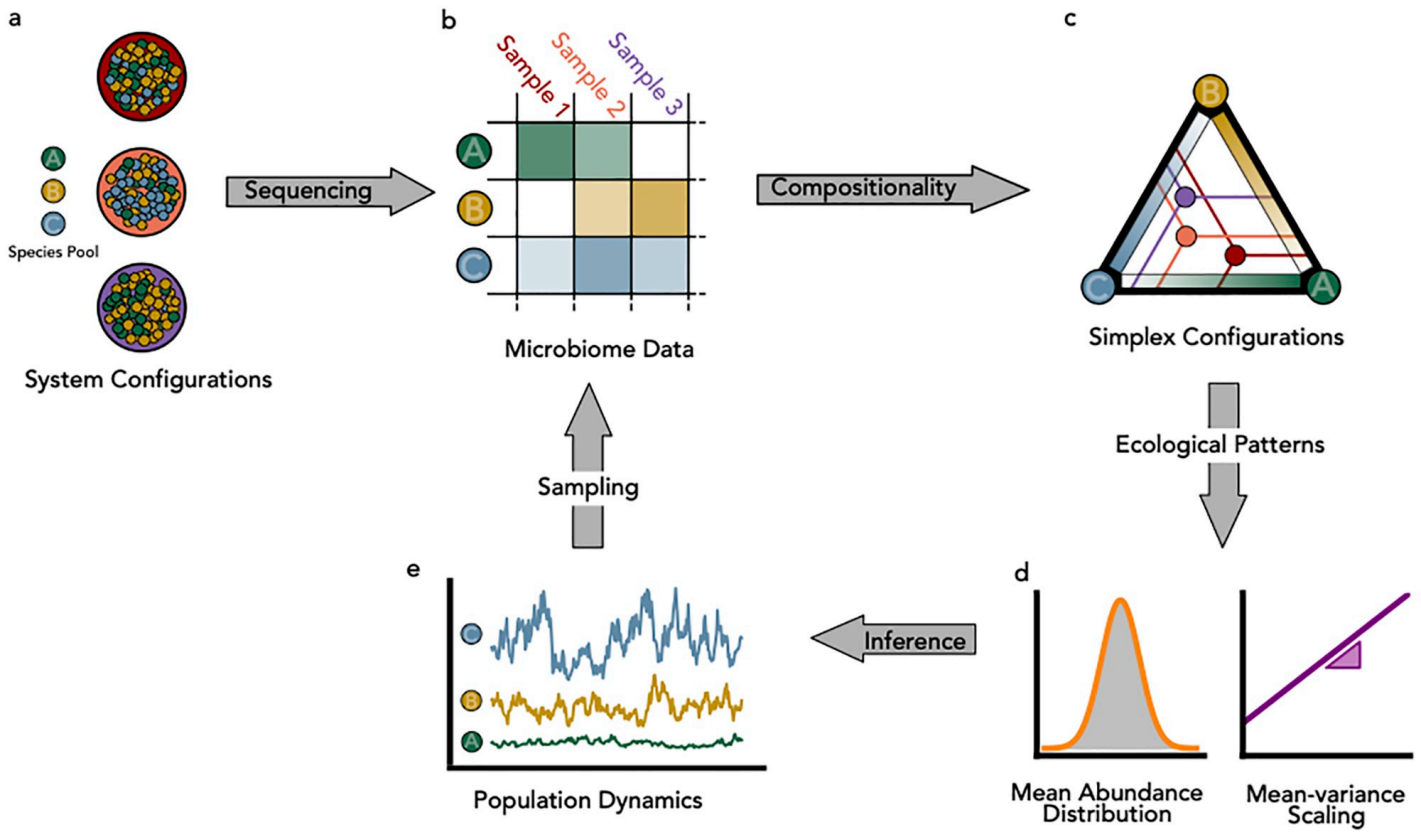
Schematic representation of the proposed theoretical framework to generate synthetic taxonomic data tables. Panel a: large coloured circles indicate different samples of microbial communities. Each small ball inside the circle represents an individual of a given species (A,B,C). We normalize the species abundances *n*_*i*_ to densities *v*_*i*_ ($\sum_{i}^{S}v_{i}=1$). Panel b: We conceptualise the empirical data generation process in terms of sequential steps that produce tabular count data. Species with low relative abundance may not be sampled, such as species B in sample 1. In panel c we show the densities phase space for the case of three species and three samples, which can be represented by a simplex. Each point within the simplex now represents one of the three samples, while each vertex represents a configuration where only the corresponding species is present in the sample. The relative abundance contribution of each species can be represented by a gradient. For a given sample, in order to obtain the relative abundance of a species, one has to project parallel to the simplex sides (as denoted by the lines). In panel d, we show the two macroecological patterns (Mean Relative Abundance distribution and the mean-variance species abundances scaling relation) that can be used to infer the parameters of the stochastic logistic growth model. Finally, in panel e, we show how the inferred population dynamics model, combined with sampling (e.g. the Multinomial Distribution), can be used to generate synthetic tabular data.

To take into account the effect of sampling, which cannot be neglected in microbiome data [13, 14], we introduce the convolution of the Dirichlet and Symmetric Scaled Dirichlet distributions with a multinomial one. In the first case, convolving the Dirichlet distribution with multinomial sampling, we obtain the Multinomial Dirichlet Distribution [19] (MD). The independent parameters of this model are thus the MAD parameters (*μ*, λ) and the TL amplitude *A*. The MD model is compositional, but it does not satisfy the TL. In the second case, by convolving the Symmetric Scaled Dirichlet distribution with the multinomial distribution, we obtain a novel model with ecologically grounded constraints (TL), which we will refer to as Multinomial Symmetric Scaled Dirichlet (MSSD). In this way, we can generate a synthetic microbiome using the relative abundance of species $\vec{v}$ as the densities and the number of reads *N* as the number of trials of the multinomial distribution (see Fig 1). In this case, the number of independent parameters to fit from the data is two, λ and *A* (or, equivalently, λ and *σ*).

We will compare the results obtained for both MD and MSSD models and also for the compositional-only-on-average stochastic logistic model originally proposed by Grilli [13], i.e., $P_{SLG}=\prod_{i=1}^{S}p_{\Gamma }\left(v_{i}|\alpha_{i},\beta_{i}\right)$, with $\sum_{i}\;{\bar{v}}_{i}=1$. To consider sampling effects and since the corresponding joint species abundance distribution does not have a sum-to-one hard constraint, we convolve *P*_*SLG*_ with the Poisson distribution and call this model the Poisson Stochastic Logistic Growth model (PSLG). In this model, the ingredient that ensures (on average) compositionality is the fact that mean abundances are constrained to sum up to one. We note that, from a statistical mechanics perspective, *P*_*ζ* = 2_ and *P*_*SLG*_ are, respectively, the microcanonical and canonical formulations of the same model. Similarly to the MD, the parameters required to fully specify the model are *μ*, λ and *A*.

Operatively, to sample from the three above described model models, we implement the following procedure (see also Fig 1): 1) Depending on the model, fit *μ*, λ, *A* from the data (for a detailed discussion about fitting procedures, see S2 Text). 2) Extract *S* average abundances from *P*_MAD_(*μ*, λ); 3) Using Eq (6) and the estimate of the coefficient *A* from the TL, generate $\vec{\beta }$ and $\vec{\alpha }$ (whose dimensions depend on *ζ*, see Eq (6)); 4) Sample the relative species abundances *v*_*i*_, *i* = 1, ..*S*_*R*_ for each of the *r* = 1…*R* samples we want to generate using Eq (7) or Eq (8); 5) For each sample, with the appropriate sampling distribution, generate species counts using relative abundances and *v*_*i*_ and the number of classified reads in the *r*−th sample, *N*_*r*_; 5) Remove all species whose relative abundance is below a given threshold *κ*. In order to understand the effect of false positive species on the patterns and models outcomes, we will apply three different cut-off *κ*_*l*_ = 4.5 × 10^−7^ (low), *κ*_*m*_ = 9 × 10^−6^ (medium), *κ*_*h*_ = 1.8 × 10^−4^ (high). The three cut-off values were obtained considering how the diversity of the dataset changes with *κ* (for a derivation of these values see Fig E in S1 File).

Equipped with these models, our aim is to investigate the statistical properties and macroecological patterns of two distinct groups of human gut microbial communities. The first is a cohort of healthy (H) individuals, while the second is a cohort of individuals affected by gastrointestinal tract diseases, which we will generally refer to as unhealthy (U).

## Microbiome metadata selection and analysis

We have selected gut microbiome data from three studies [4, 10, 27], where both sequencing data and sample metadata are available for controls and three gastrointestinal tract diseases: Crohn’s Disease, Ulcerative Colitis and Inflammatory Bowels Syndromes. In the following, we will refer to the controls as the H group and all samples from pathological conditions as the U group. We have filtered so as to have a homogeneous and not biased dataset (see S1 Data for details). In general, we have selected patients who were less affected (at least at the time of the study) by medical treatments, to limit the impact of different drug treatments on the gut microbiome. After this filtering procedure, we ended up with *R*_*H*_ = 91 shotgun metagenomic samples from healthy control individuals and *R*_*U*_ = 202 samples from dysbiotic microbiomes.

We have implemented (for details, see S3 Text) a computational pipeline to process throughput sequencing metagenomic data following best practices [32], such as quality filtering (removing reads with *Q* < 20) and human DNA decontamination using the NCBI human genome assembly (GRCH38).

The metagenomic taxonomic profiling tool we have adopted in our analysis is the *Kaiju* classifier [26], which converts metagenomic reads in all possible open reading frames and searches for the best match in a protein database. The advantage of this approach is that, due to the degeneracy of the genetic code, it is robust to random mutations along the genome and, as such, to evolutionary divergences between the dataset and the reference catalogue of genomes. As a reference species catalogue, we have used RefSeq [33], which contains protein sequences from complete archaeal and bacterial genomes. Metagenomic samples were profiled on October 13th 2020. Eventually, we have classified (on average across samples) 39% of reads in H samples and 37% U, at the species level. From these we build two data-tables, one for each of the two classes (H and U), having the different species (S) as rows and the samples (R) as columns. Each {*i*, *j*} entry gives the corresponding relative species abundances *v*_*i*_ of the sample *j*, so that $\sum_{i=1}^{S}v_{i}^{j}=1$. Relative abundances are obtained by dividing the number of reads assigned to a given species by the total number of reads recognized at the species level for that sample. To implement the relative abundance threshold, we set to zero all species abundances less than the relative abundance cut-off *κ*, i.e. $v_{i}^{j}=0$ if $v_{i}^{j}<\kappa$.

## Results

For ease of reference, Table 1 provides definitions for all the used acronyms, see also S1 Table.

**Table 1:** List of abbreviations and their descriptions.

Abbrev.	Description
H, U	Healthy, Unhealthy
OTU	Operational Taxonomic Unit
MAD	Mean Abundance Distribution
TL	Taylor’s Law
SAD	Species Abundance Distribution
AO	Abundance-Occurrence
SAR	Species-Area Relation
BIC	Bayesian Information Criterion
PSLG	Poisson Stochastic Logistic Growth, Model
MD	Multinomial Dirichlet, Distribution
MSSD	Multinomial Symmetric Scaled Dirichlet, Distribution

### Mean abundance distribution and Taylor’s law

**Fig 2:**
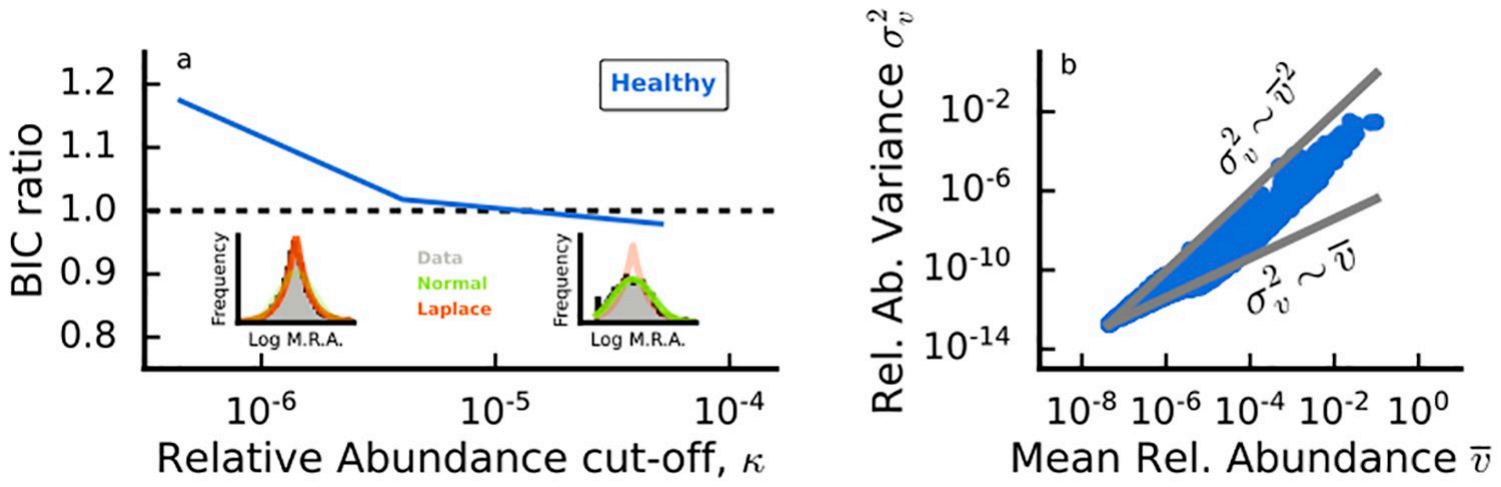
Panel a: *Mean Abundance Distribution* shape displays a dependence on the relative abundance cut-off *κ*. The BIC ratio curve for healthy and disease-related data collapse onto the same line. Panel b: Taylor’s Law holds in empirical human gut communities, both in health and in disease. For rare species, it is difficult to discriminate between Poisson-like and Taylor-like scaling, due to the fact that rare species are present only in a few samples. For simplicity, we report the scatter plot only in the healthy case. A brief discussion of the fitting procedure can be found in S2 Text.

We find that a similar *P*_*MAD*_ is observed in both the H and U dataset, and its shape depends on the relative abundance cut-off *κ*. In particular, for *κ* < 10^−5^ the MAD displays a *Log-Laplace* shape, i.e. $P_{\text{MAD}}\left(\bar{x}|\mu ,\lambda \right)=e^{-\frac{|\text{log}\;\bar{x}-\mu |}{\lambda }}/\left(2\lambda \bar{x}\right)$, while for *κ* > 10^−5^ the MAD is a Log-Normal distribution $P_{\text{MAD}}\left(\bar{x}|\mu ,\lambda \right)=e^{-\frac{{\left(\text{log}\;\bar{x}-\mu \right)}^{2}}{2\lambda^{2}}}/\left(\bar{x}\sqrt{2\pi \lambda^{2}}\right)$, the same found for OTU 16s data [13]. These distributions indicate a high heterogeneity in mean abundances having both heavy tails. On the y-axis of Fig 2 we show the Byesian Information Criterion (*BIC*) ratio: if it is greater than one, it indicates that the Laplace distribution is a better fit than the Log-Normal, while if *BIC*<1, then the opposite is true. We thus obtain the values of *μ* and λ for three different thresholds of *κ*. As can also be seen from Fig 2, the Laplace distribution is usually a better description of the MAD, except for a large threshold where the MAD clearly displays a Log-Normal shape (and compatibly with the OTU case [13]). In particular, by fitting *P*_MAD_ we find λ_*H*_ = 1.407 ± 0.005 and λ_*U*_ = 1.413 ± 0.007 (uncertainty of the fit evaluated with a bootstrap procedure, for details see S2 Text).

Regarding the TL, we find that the value of inferred *A* depends on the threshold *κ*. On the other hand, the exponent *ζ* ≈ 2 is remarkably robust for all *κ* and for H and U samples. In particular, for each value of *κ* we compare the *R*^2^-score ratio of the best fit power-law to that with fixed *ζ* = 2 finding $\frac{R_{\zeta =2}^{2}}{R_{fit}^{2}}\approx 1$ suggesting a negligible discrepancy between the two models for this scaling relation. Therefore, in the following, we assume *ζ*_*Data*_ = 2.

### Emergent ecological patterns in healthy and unhealthy microbiomes

In this section, we investigate macro-ecological emergent patterns in gut microbiomes, and test which model can describe them, and whether there are any statistically significant deviations in such patterns between H and U samples.

We will focus on the following ecological patterns of the gut microbiomes: 1) *α* and *γ* diversity [34], defined as the number of different species in each local community (i.e., samples) and H and U meta-communities (i.e. union of all H/U samples), respectively; 2) The abundance-occupancy distribution, describing the probability that a species with mean relative abundance $\bar{v}$ is found in a fraction $\bar{o}$ of the total number of samples within a meta-community; 3) The species abundance distribution (SAD) of the H and U meta-communities; 4) The relation between the number of species observed in a local community normalized to the meta-community one (*α*/*γ*-diversity, also known as Whittaker’s beta diversity) and its sequencing depth (i.e., the metagenomic version of the species-area relationship).

To compare a given ecological pattern obtained from the data with the corresponding one produced by a model, we first set the scaling exponent *ζ* (*ζ* = 2 for MSSD and SLG, *ζ* = 1 for MD). Second, we fit the parameters *μ*, λ, *A* (in the case of MSSD, due to model symmetries and as explained in S2 Text, fitting *μ* is not necessary) from the data with no cut-off (*κ* = 0). We then generate 500 realizations of the two meta-communities (H and U) with the same number of reads (*N*), of species (*S* = *γ*), and of samples *R* as found in the data. Eventually, we consider the three relative abundance cut-offs *κ* both in the empirical and simulated data (i.e., all species with *v*_*i*_ < *κ* are set to zero) and for each pattern we calculate a *R*^2^-like score (see S1 Text). The final *R*^2^-score we assign to the model is the average of all instances of the model.

**Fig 3:**
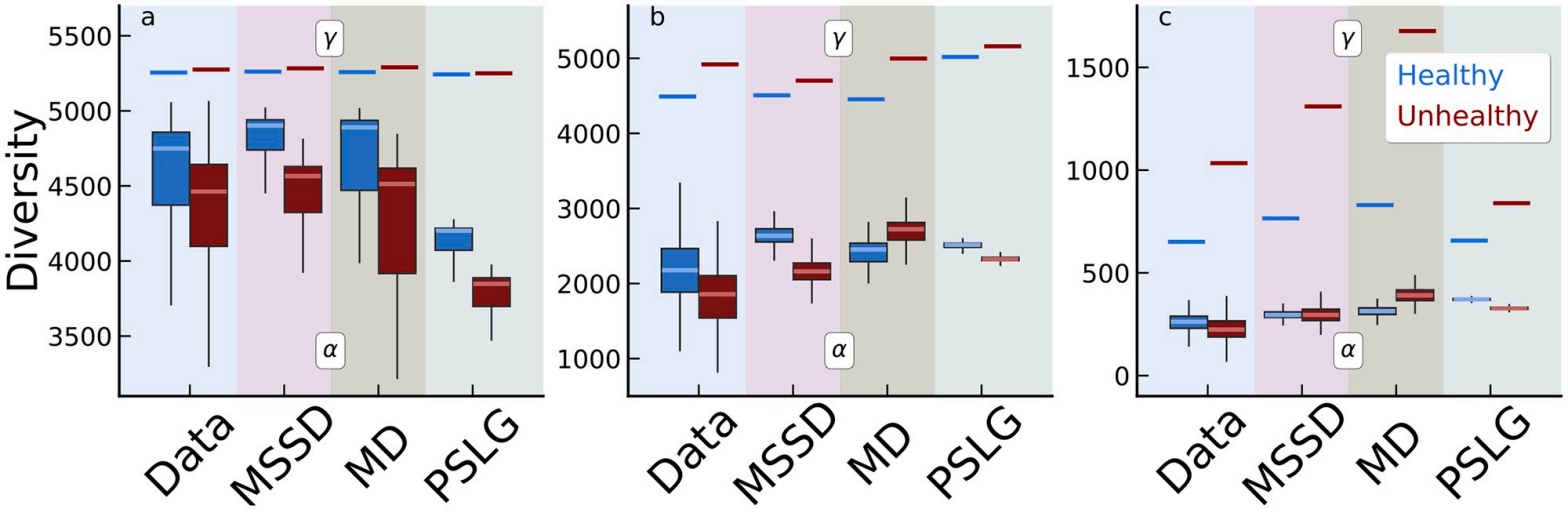
Box-whiskers plots describe the average local (*α*) diversity, while horizontal bars indicate the corresponding metacommunity (*γ*) diversity. In healthy gut microbiomes (in blue) we find higher average *α* and lower *γ* diversity than unhealthy ones (in red). The three panels represent different threshold relative abundance cut-off *κ*: a) low (*κ*_*l*_ = 4.5 × 10^−7^); b) medium (*κ*_*m*_ = 9 × 10^−6^); c) high (*κ*_*h*_ = 1.8 × 10^−4^). In each panel, we compare the diversity of the empirical H and U diversity with respect to the one generated by three different null models: MSSD, SLG, and MD. MSSD is found to be the best model, especially for low and medium *κ*.

The first patterns we consider are the *γ* and *α* diversities. The values of such quantities are strongly dependent on *κ*, but reveal a persistent qualitative regularity among the three regimes. Indeed, the overall (*γ*) diversity of species found in the unhealthy meta-community is larger than that found in healthy microbiomes (*γ*_*H*_ < *γ*_*U*_) for all *κ*. On the other hand, the *average* local diversity ($\bar{\alpha }$) found for the H samples is larger compared to that of the U samples, i.e., (${\bar{\alpha }}_{H}>{\bar{\alpha }}_{U}$) (see Fig 3).

**Fig 4:**
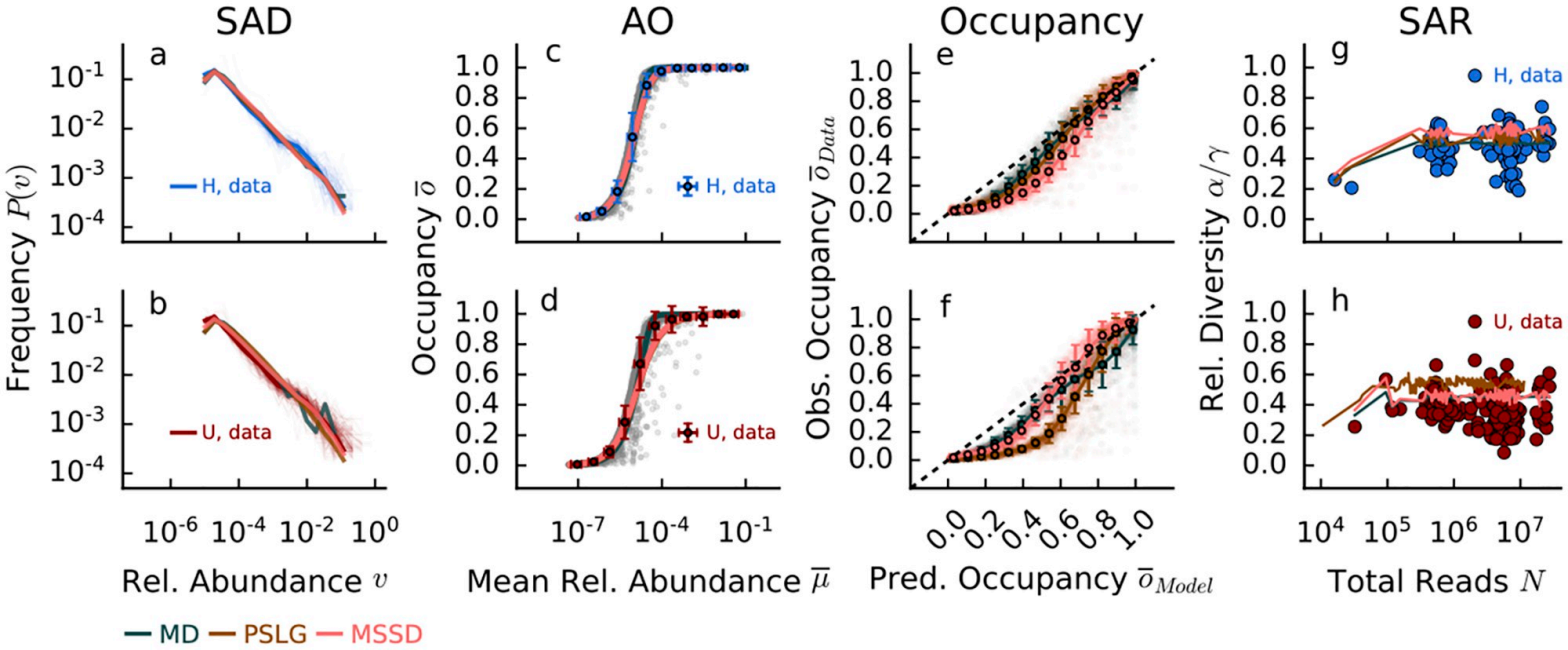
Comparison of emergent empirical ecological patterns in healthy (H, top panels) and unhealthy (bottom panels) microbiomes and for MSSD, SLG and MD models. Panels a-b) Species Abundance distribution (SAD); c-d) Species abundances occupancy curve (AO). Grey shaded points refer to single species mean relative abundance and occupancy; e-f) Empirical vs. predicted occupancy curves (O). Shaded points refer to the predicted/observed occupancy of single species. To compare the occupancy of simulated and observed species, we sort the simulated species according to their mean abundance; g-h) Species Area Relationship (SAR) curves. These patterns can be investigated for different cut-offs (see Fig A-D S1 File), here we show the average threshold cut-off *κ*_*m*_ = 9 × 10^−6^.

The second pattern we consider is the SAD [25], which describes, in a given sample the probability of observing species with a given abundance. In agreement with previous results obtained with 16s OTU data [13, 35] and also with shotgun data [36], we find that the SAD displays a heavy tail, that is compatible with small and medium cut-off with power-law distributions with exponents around 1.7 (see Fig G in S1 File for more details). For large thresholds, the SAD is more compatible with a Log-Normal distribution. No significant differences are observed between the H and U individuals (see Fig 4a). Moreover, all the models (PSLM, MSSD, and MD) generate SADs that are compatible with the empirical ones. These results confirm [37, 38] that SADs are not informative patterns of the underlying ecological mechanisms driving species abundances.

We then investigate the relation between the log-mean relative abundance of a species and its occupancy, which we refer to as the abundance-occupancy (AO) curve. We have already introduced the average relative abundance of species *i* as ${\bar{v}}_{i}=\frac{1}{R}\sum_{j=1}^{R}v_{i}^{j}$, while we define the *occupancy* of species *i* as ${\bar{o}}_{i}=\frac{1}{R}\sum_{j=1}^{R}\theta \left(v_{i}^{j}\right)$, where *θ* is the Heaviside Theta, which converts relative abundance data into presence/absence ones. The relation between $\bar{v}$ and $\bar{o}$, as shown in Fig 4b, describes how likely it is for a species, given its average relative abundance, to be sampled in a realization of the system. This relation is also known as intensity-sparsity relation [14]. When the low/medium value of *κ* is set, the curve suddenly saturates, suggesting that a large proportion of the available *S* = *γ* species are expected to be sampled. In this scenario, the community is dominated by rare species, and thus almost all sampled individuals belong to different species, thus saturating the diversity very fast. As *κ* increases, we have fewer and fewer species that are rare in relative abundance but, at the same time, are harder to sample. As Fig 4 shows (panels c, d), this behavior is common to all models and thus does not discriminate against the underlying ecological processes. However, for low (high) *κ* the MD typically underestimates (overestimates) the occupancy of species (for details see Fig A in S1 File).

We can also directly compare the occupancy curves obtained from the presence-absence data with those predicted by the models (as shown in Fig 4e and 4f). Interestingly, contrary to the previous case, here there are differences in how well the models describe the empirical emergent patterns. In particular, for H samples, the PSLG model outperforms the MSSD one, whereas MSSD better predicts the pattern of U samples. Such results and goodness of fit for the MD model are not robust to different thresholds (see Table 2 and Fig A in S1 File).

**Table 2:** *R*^2^ scores for the considered macroecological patterns and for three different thresholds: Low *κ*_*l*_, medium *κ*_*m*_ and high *κ*_*h*_.

		AO Curve			Species Occupancy			SAR Curve		
		*κ*_*l*_	*κ*_*m*_	*κ*_*h*_	*κ*_*l*_	*κ*_*m*_	*κ*_*h*_	*κ*_*l*_	*κ*_*m*_	*κ*_*h*_
H	MSSD	0.997	0.996	0.983	0.850	0.823	0.830	0.819	0.695	-15.131
	PSLG	0.998	0.995	0.983	0.908	0.912	0.811	0.984	0.585	-22.655
	MD	0.937	0.995	0.944	0.516	0.925	0.337	0.776	0.005	-59.763
U	MSSD	0.992	0.982	0.987	0.885	0.861	0.776	0.976	0.840	-0.779
	PSLG	0.987	0.982	0.984	0.911	0.571	0.569	0.988	0.522	-1.244
	MD	0.902	0.993	0.864	0.562	0.872	0.485	0.874	0.726	-1.302

Finally, we consider the “metagenomic” version of the species-area-relation (SAR) [39], i.e., how the diversity increases with increasing sampled area. Here, the area is substituted by the total number of classified reads. Therefore, we consider increasing the number of reads combining the samples of each group and calculate the normalized diversity as the number of unique species in the aggregated community divided by the *γ* diversity. Although all models on average slightly overestimate the overall diversity, in H samples they all perform similarly, while for U samples MSSD and MMD models (which can generate the same number of total reads as found in the data) outperform the SLG model.

Table 2 summarizes all the results and the comparison between the goodness of fit (measured as explained in S1 Text) of the three models for the presented emergent empirical ecological patterns of gut microbiomes in both health and disease states.

**Fig 5:**
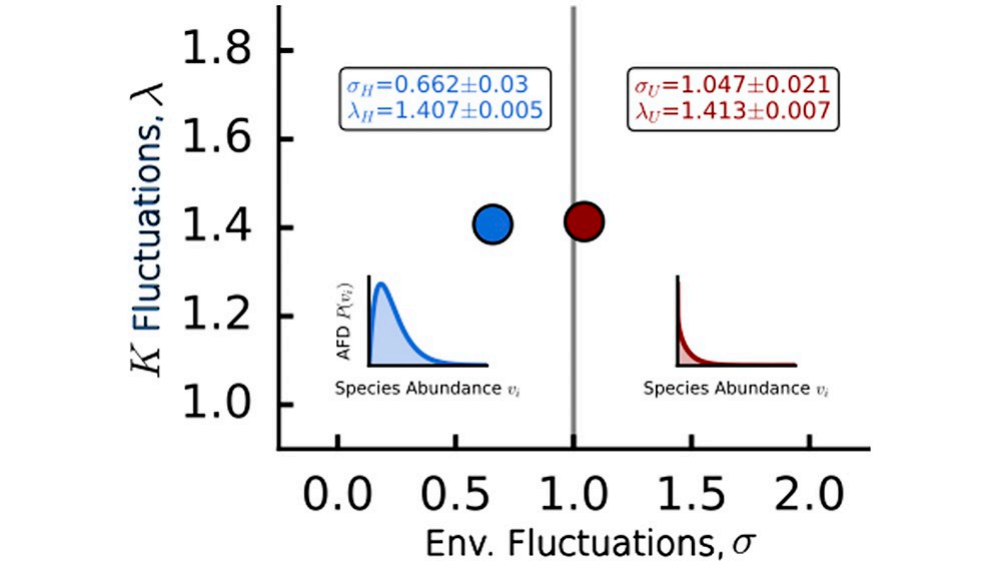
The parameters of Taylor’s law and mean abundance distribution are informative about the underlying stochastic logistic model. The values obtained from the data suggest that healthy and unhealthy species abundance distribution follow different qualitative behaviors, with the unhealthy case being prone to more extinction. Average and standard deviation estimates of the parameters have been obtained through a bootstrap procedure.

Finally, by fitting the MSSD, we can gain insight into possible differences in species population dynamics within microbiomes of healthy and diseased individuals. As reported in Fig 5, by fitting the MAD we find λ for both H and U cohorts. We can interpret it as the fluctuation scale of the carrying capacities. This turns out to be statistically indistinguishable between the H and U cohorts. However, the most relevant difference comes from the value we infer for *σ*, the width of the environmental fluctuations. The stochastic logistic growth model Eq (1) predicts that the abundance fluctuations distribution has a polynomial part with an exponent greater than zero if *σ* > 1 (as observed in the unhealthy cohort) and less than zero if *σ* < 1 (as observed in the healthy cohort).

## Discussion and conclusion

By inferring the parameters of the models that best describe these patterns, we have obtained ecological insights into dysbiosis that would not be directly accessible from the data. In particular, we have found that while the intrinsic logistic population dynamics are similar in the H and U cohorts (they have very similar carrying capacities determined by λ), fluctuations in growth rates due to extrinsic environmental factors (given by *σ*) are much stronger in the U microbiomes. This is reflected in the abundance fluctuations distribution shifting from a modal distribution in H samples to a power-law one (with exponential cut-off) in U microbiomes, i.e., the probability of a species being very rare is higher in dysbiosis.

This result allows us to explain why in the H cohorts we observe a higher *α*, but a lower *γ* diversity: the species in the U microbiomes experience higher fluctuations, and are thus more prone to local extinctions, but are also subject to higher turnover (thus increasing the global diversity of the group). Therefore, we believe that for dysbiois the celebrated Anna Karenina principle “All happy families are alike; each unhappy family is unhappy in its own way” (Lev Tolstoj, Anna Karenina, 1877) also holds: ‘All healthy gut microbiomes are alike; each unhealthy gut microbiome is unhealthy in its own way”. In fact, there are convergent observations suggesting that dysbiosis can be attributed to host-specific factors [40, 41]. We also have implemented a stratification analysis (see Fig A-C in S2 File), where we investigated whether the distinct diversity patterns for healthy and unhealthy microbiomes also hold if only specific gastrointestinal diseases are considered. We have observed that while Chrohn’s disease (CD) and ulcerative colitis (UC) exhibit trends consistent with our general findings (lower average *α* diversity and higher *γ* diversity in U patients), inflammatory bowel syndrome (IBS) presents less systematic tendencies. This distinction is noteworthy given the clinical challenges associated with the diagnosis of IBS. We have also performed a stratified inference of the environmental noise (*σ*) and carrying capacity heterogeneity (λ) parameters, finding results compatible with the previous ones. For the different cohorts, there is no substantial difference in λ; On the other hand, we have found that CD and UC are associated with an environmental noise strength *σ* close to one and larger than healthy microbiomes, supporting our interpretation discussed above. Again, IBS has a behavior more similar to that of healthy individuals.

We have also shown that microbiome species abundance data exhibit a Taylor law with a *ζ* value of 2. Interestingly, the MD model, by its design, does not satisfy this fundamental constraint. However, as shown in Table 2, the MD model is capable of generating AO curves and SADs that are compatible with the data at any threshold. Similarly, it can accurately reproduce Occupancy curves at a medium threshold and SAR curves at a low threshold. This finding highlights that not all patterns and thresholds are equally informative. Some are more effective than others in differentiating between models and underlying ecological processes. Although AO curves have previously been used to test specific underlying ecological theories [42], our results suggest that the shape of the AO curve is simply the result of two main ingredients: heterogeneous population averages and random sampling. Similarly, all patterns at low thresholds are dominated by a large number of rare species (which in shotgun data are probably false positives [15]).

Something similar occurs for the SAD, where all models are practically indistinguishable from one another. Indeed, the fact that different models (i.e., processes) can lead to very similar SAD patterns has long been known in theoretical ecology [25, 43].

For the SAR curves there is a very strong impact of the thresholds on the models goodness of fit. At low thresholds, all models performed well, thus not providing any discriminatory power for the right choice of the model. However, at the high threshold, there was a significant drop in the goodness of fit for all models. In fact, due to removal of all the rare species, the SAR loses its characteristic shape, and thus it is not useful for models comparison. At the intermediate threshold, the MSSD model performed the best, although it is important to note that the *R*^2^ value is lower compared to its maximum *R*^2^ = 0.819. In this case, we also have found that all models fit U samples better than H samples. This effect is probably because in U samples the *γ* diversity is higher, and we have many more rare species, thus increasing the slope of the SAR (that is, in general, overestimated by the models).

Upon closer examination of the Species Occupancy patterns in Table 2, notable differences emerged between healthy and unhealthy samples at the medium threshold. In the H samples, the SLG model showed the highest goodness of fit, closely followed by the MSSD model. On the contrary, the unhealthy samples showed a different pattern. The SLG model, which performed strongly in the healthy samples, showed a marked decrease in its goodness of fit, indicating potential challenges in capturing the complexity of species occupancy in unhealthy systems at this threshold. The MSSD model also showed a reduction in performance, but remained relatively more consistent compared to the SLG model. The performance of the MD model for both H and U samples was extremely variable, depending on the cut-off *κ*. For the medium threshold, the fit—although not as good as the one of the MSSD and SLG models—had a relatively high *R*^2^, whereas for the low and high thresholds, it decreased markedly.

In our study, we thus have found that, unlike to AO curves and SADs, species occupancy and diversity curves provide key insights into the performance of various models. Models incorporating Taylor’s law with *ζ* = 2 (SLG and MSSD) offer a better explanation of the data at the medium threshold (which has proven to be the most informative cut-off for false positives). This suggests that large species fluctuations, as dictated by Taylor’s law with a scaling exponent of *ζ* = 2, are important for accurately predicting presence/absence patterns and species diversity in empirical datasets. We also have found that models that perform well in healthy communities may not necessarily do so in unhealthy ones, and vice versa. This insight is crucial for ecological modeling and could guide future research in developing or choosing models that are tailored to the specific conditions of the ecological systems being studied. Furthermore, we have found that the SLG model, although effectively similar to the MSSD in many respects, underestimates species occupancy (see Fig 4f) and overstimates species diversity (see Fig 4g). The reason is that SLG can generate ecological communities with a number of individuals that is only on average as the one of the corresponding sampled data, while MSSD implements strict compositionality of the data.

All in all, we suggest that although compositionality and sampling strongly obscure ecological signals, making most empirical patterns qualitatively similar, there are indeed quantitative ecological differences between microbial communities of the gut microbiome in health and disease. In particular, considering only a few relevant patterns like Taylor’s law and species occupancy and using interpretable analytical models that also include environmental noise, we can propose an interpretation of the observed differences in the taxonomic data, eventually shedding light on the underlying ecological processes characterizing informative emergent patterns, such as the specific trend of *α* and *γ* diversity in both H and U cohorts. Thus, we conclude that dysbiosis is characterized by stronger turnover than healthy microbiomes, which is due to larger environmental fluctuations.

## Supporting information

S1 Table List of abbreviations. (PDF)

S1 Data Data selection, processing and analysis. (PDF)

S1 File Supplementary figures. (PDF)

S2 File Stratification analysis. (PDF)

S1 Text Model testing. (PDF)

S2 Text Supplementary methods. (PDF)

S3 Text Metagenomic pipeline implementation. (PDF)
