# Supplementary material for: Emergent ecological patterns and modelling of gut microbiomes in health and in disease
Source: PLoS Comput Biol. 2024 Sep 27;20(9):e1012482. doi: 10.1371/journal.pcbi.1012482 (PMC11493414; doi:10.1371/journal.pcbi.1012482)
Supplement: S1 Table — (PDF) [file pcbi.1012482.s001.pdf]

Emergent Ecological Patterns and Modelling of Gut Microbiomes in Health and in Disease: S1 Table

J. Pasqualini[1,*], **S. Facchin**[2], **A. Rinaldo**[3,4], **A. Maritan**[1], **E. Savarino**[2], **S. Suweis**[1,*]

[1] *Dipartimento di Fisica "G. Galilei" e INFN sezione di Padova, University of Padova, Padova, Italy*

[2] *Dipartimento di Scienze Chirurgiche, Oncologiche e Gastroenterologiche (DiSCOG), University of Padova, Padova, Italy*

[3] *Dipartimento di Ingegneria Civile, Edile e Ambientale (ICEA), University of Padova, Padova, Italy*

[4] *Laboratory of Ecohydrology, École Polytechnique Fédérale Lausanne, Lausanne, Switzerland*

S1 Table: Abbreviations

- H,U: healthy, unhealthy
- OTU: operational taxonomic unit
- MAD: mean abundance distribution
- TL: Taylor's Law
- SAD: species abundance distribution
- AO: abundance-occurrence
- SAR: species-area relation
- BIC: Bayesian Information Criterion
- PSLG: Poisson stochastic logistic growth, model
- MD: multinomial Dirichlet, distribution
- SDD: scaled Dirichlet, distribution
- MSSD: multinomial symmetric scaled Dirichlet, distribution
