# Supplementary material for: Emergent ecological patterns and modelling of gut microbiomes in health and in disease
Source: PLoS Comput Biol. 2024 Sep 27;20(9):e1012482. doi: 10.1371/journal.pcbi.1012482 (PMC11493414; doi:10.1371/journal.pcbi.1012482)
Supplement: S1 Data — (PDF) [file pcbi.1012482.s002.pdf]

Emergent Ecological Patterns and Modelling of Gut Microbiomes in Health and in Disease: S1 Data

J. Pasqualini[1,*], S. Facchin[2], A. Rinaldo[3,4], A. Maritan[1], E. Savarino[2], S. Suweis[1,*]
[1] *Dipartimento di Fisica "G. Galilei" e INFN sezione di Padova, University of Padova, Padova, Italy*
[2] *Dipartimento di Scienze Chirurgiche, Oncologiche e Gastroenterologiche (DiSCOG), University of Padova, Padova, Italy*
[3] *Dipartimento di Ingegneria Civile, Edile e Ambientale (ICEA), University of Padova, Padova, Italy*
[4] *Laboratory of Ecohydrology, École Polytechnique Fédérale Lausanne, Lausanne, Switzerland*

S1 Data: Meta Data selection, processing and analyses

We collected our data from three metagenomic gut microbiome studies [1], [2], [3]. In this section, we outline the procedure we followed to build this curated dataset from both a quantitative and literature review perspective.

The idea here is to assign an accession code to each patient, which we will use to download raw metagenomic sequence data from the SRA/ENA online portal. Once the accession list was obtained, we wanted to associate each accession code with a label that would tell us what clinical condition the patients were suffering from. We will build a dataset in which each patient will be associated with two objects, namely features and targets, with the following structure.

First, we established reasonable criteria to make our aggregated cohort as homogeneous as possible. To filter the vast literature available on human gut metagenomics, we only considered studies that met the following criteria

- Whole genome shotgun sequencing
- Age between 15 and 65
- No ongoing therapy at sampling date
- Available metadata of clinical/demographic records

The studies we found and employed in our meta-analysis were the following:

- Franzosa, E.A., Sirota-Madi, A., Avila-Pacheco, J. et al. *Gut microbiome structure and metabolic activity in inflammatory bowel disease*. [3]
- Ruben A.T. Mars, Dan Knights, Purna C. Kashyap et al *Longitudinal Multi-omics Reveals Subset-Specific Mechanisms Underlying Irritable Bowel Syndrome* [1]
- Lloyd-Price, J., Arze, C., Ananthakrishnan, A.N. et al. *Multi-omics of the gut microbial ecosystem in inflammatory bowel diseases* [4]

We scraped from two sources of metadata: the **fastq**-related metadata, usually available on the experiment page of the online database (SRA/ENA), and files from the supplementary material of the studies considered. Typically, all links and information were provided in the data availability statement of the papers. This division of metadata resources forced us to carefully integrate metadata, as it was not easy to assign all metagenomic samples to each patient. An important step was to find a variable that allowed us to merge the different metadata tables. Furthermore, non-standardisation of metadata is imposed to do this on a case-by-case basis.

The first criterion S1 is a requirement for the study to be included. With regard to age, when the paediatric sub-cohort was included in the study, the age of the patients was usually provided, allowing for more precise filtering. If there was no explicit information on age, only cohorts compatible with our range were considered.

The third criterion was more stringent and difficult to assess. In one study, clinical metadata were not provided, but recruitment criteria were available in the paper and were consistent with those we adopted [1]. With the clinical metadata provided, our selection strategy was to aggregate the different characteristics reported. Taking into account the treatment characteristics (e.g. antibiotic treatment, immunosuppressant, previous bowel surgery, etc.), we created a new one called *treatment*. If a sample related to a given patient was under any treatment at the time of sampling, it

was discarded. In addition, as two out of three studies were longitudinal, we only considered samples at baseline and followed the same inclusion criteria. As a schematic example, we can write

Sample	Immunosuppressants	Surgery	Treatment
SRR6468505	NO	NO	NO
SRR6468557	NO	YES	YES
ERR4775890	YES	NO	YES

After determining how to recruit samples from the hundreds of candidates, we ended up with 293 final samples. For each of these, we obtained its associated metagenomic run and performed pre-processing and taxonomic classification, as discussed in the main text. At the end of this process, we were able to obtain a dataset in which we were able to assign diagnostic metadata to each sample, the ones were mainly focused on in the main text.

S1.1 Franzosa et. al, 2019

This study involved two cohorts, one for discovery ($n_d = 155$) and one for validation ($n_v = 65$) of CD, UC and non-IBD controls [4].

The discovery cohort consisted of individuals enrolled in PRIMS (the Prospective Registry in IBD Study at Massachusetts General Hospital). This discovery cohort included 155 subjects: 68 with Crohn's disease (CD), 53 with ulcerative colitis (UC) and 34 non-IBD controls.

The NLIBD validation cohort consisted of 65 subjects enrolled in two different studies in the Netherlands. 22 controls were enrolled in the LifeLines-DEEP general population study. 43 subjects with IBD were enrolled in a study at the Department of Gastroenterology and Hepatology, University Medical Center Groningen (UMCG) [5].

Metadata for the experiments was obtained by linking to the online version of the article. In particular, the metadata used in our work can be found at in Table 4. From this file, we kept only the first 10 columns, which contain information such as SRA metagenome name, age, diagnosis and treatment. We will refer to this file as **franzosa_metadata.csv**.

Raw reads metadata was collected using the SRA platform where are all available with the BioProject code PRJNA400072.

S1.2 Mars et al., 2020

This study was conducted exclusively at the Mayo Clinic Rochester. Participants were recruited through Mayo Clinic Institutional Review Board (IRB)-approved advertisements and represent an IBD cohort aged 18-65 years. Participants were given the option to undergo two flexible sigmoidoscopies [1].

Volunteers with a history of abdominal surgery, previous inflammatory conditions, antibiotic therapy, bleeding risk, pregnancy, being a vulnerable adult, and age under 18 or over 65 years were excluded. In addition, subjects with other diseases, conditions, or habits that would interfere with the completion of the study were excluded. Additional information about the subjects was collected, such as medical records and physiological characteristics. As reported in this description, the recruitment criteria were compatible with ours, and relative metadata are not fundamental to our filtering.

The metadata associated with the sequencing run is available on the ENA page of the experiment with the accession code PRJEB37924. This file was useful to establish the association between sample and diagnosis, as each sample carried information about the patient identifier.

As reported in the paper, additional metadata can be found on the relative Mendeley page. We downloaded the first file (25MB) and used the last table of **biopsy_id_map** to distinguish between healthy and IBS patients. More specifically, the labels allowed us to distinguish between IBS-C (constipation) and IBS-D (diarrhoea), but due to the small cohort size we decided to identify all patients as IBS. This table not only allowed us to assign a diagnostic label to each patient, but also to construct a time series of their stool samples. As suggested in the article, the feature **ID on Tube** was used as a merging variable between metadata sources.

S1.3 Lloyd-Price et al., 2019

This study was part of the Integrative Microbiome Project, the second iteration of the Human Microbiome Project. Five medical centres participated in the IBDMDB: Cincinnati Children's Hospital, Emory University Hospital, Massachusetts General Hospital, Massachusetts General Hospital for Children, and Cedars-Sinai Medical Center [3]. Patients were

approached for potential recruitment if they presented for routine age-related colorectal cancer screening, evaluation for other gastrointestinal (GI) symptoms, or suspected IBD with either positive imaging (e.g., colonic wall thickening or ileal inflammation) or symptoms of chronic diarrhea or rectal bleeding. Potential participants were excluded if they were unable or unwilling to provide tissue, blood or stool samples, were pregnant, had a known bleeding disorder or acute gastrointestinal infection, were being actively treated with chemotherapy for malignancy, had a diagnosis of indeterminate colitis, or had undergone previous gastrointestinal surgery.

An initial colonoscopy was performed at enrolment. Subjects without a diagnosis of IBD were classified as 'non-IBD' controls.

This creates a control group that, although not completely 'healthy', differs from the IBD cohorts specifically by clinical IBD status. Differences observed between these groups are therefore more likely to be IBD-specific differences, rather than differences due to general GI distress. A total of 132 subjects participated in the study.

Descriptions of each participant and sample were recorded at baseline and at each sample collection. At baseline (i.e., during or before the screening colonoscopy), subjects completed a questionnaire on reported symptoms, the Short Inflammatory Bowel Disease Questionnaire, a food frequency questionnaire, and an environmental questionnaire.

We collected clinical metadata from the portal https://ibdmdb.org, where clinical information about subjects, such as treatment and diagnosis, was available. As we downloaded the entire project metadata, we filtered to keep only stool metagenomic samples. The treatment variables in this case were antibiotics, chemotherapy, immunosuppressants. Raw read metadata was collected on the corresponding ENA page with the experiment accession code PRJNA398089.

References

[1] Ruben AT Mars, Yi Yang, Tonya Ward, Mo Houtti, Sambhawa Priya, Heather R Lekatz, Xiaojia Tang, Zhifu Sun, Krishna R Kalari, Tal Korem, et al. Longitudinal multi-omics reveals subset-specific mechanisms underlying irritable bowel syndrome. *Cell*, 182(6):1460–1473, 2020.

[2] The integrative human microbiome project. *Nature*, 569(7758):641–648, 2019.

[3] Jason Lloyd-Price, Cesar Arze, Ashwin N Ananthakrishnan, Melanie Schirmer, Julian Avila-Pacheco, Tiffany W Poon, Elizabeth Andrews, Nadim J Ajami, Kevin S Bonham, Colin J Brislawn, et al. Multi-omics of the gut microbial ecosystem in inflammatory bowel diseases. *Nature*, 569(7758):655–662, 2019.

[4] Eric A Franzosa, Alexandra Sirota-Madi, Julian Avila-Pacheco, Nadine Fornelos, Henry J Haiser, Stefan Reinker, Tommi Vatanen, A Brantley Hall, Himel Mallick, Lauren J McIver, et al. Gut microbiome structure and metabolic activity in inflammatory bowel disease. *Nature microbiology*, 4(2):293–305, 2019.

[5] Jingyuan Fu, Marc Jan Bonder, María Carmen Cenit, Ettje F Tigchelaar, Astrid Maatman, Jackie AM Dekens, Eelke Brandsma, Joanna Marczynska, Floris Imhann, Rinse K Weersma, et al. The gut microbiome contributes to a substantial proportion of the variation in blood lipids. *Circulation research*, 117(9):817–824, 2015.
