# Supplementary material for: Emergent ecological patterns and modelling of gut microbiomes in health and in disease
Source: PLoS Comput Biol. 2024 Sep 27;20(9):e1012482. doi: 10.1371/journal.pcbi.1012482 (PMC11493414; doi:10.1371/journal.pcbi.1012482)
Supplement: S1 File — (PDF) [file pcbi.1012482.s003.pdf]

Emergent Ecological Patterns and Modelling of Gut Microbiomes in Health and in Disease: S1 File

J. Pasqualini[1,*], **S. Facchin**[2], **A. Rinaldo**[3,4], **A. Maritan**[1], **E. Savarino**[2], **S. Suweis**[1,*]
[1] *Dipartimento di Fisica "G. Galilei" e INFN sezione di Padova, University of Padova, Padova, Italy*
[2] *Dipartimento di Scienze Chirurgiche, Oncologiche e Gastroenterologiche (DiSCOG), University of Padova, Padova, Italy*
[3] *Dipartimento di Ingegneria Civile, Edile e Ambientale (ICEA), University of Padova, Padova, Italy*
[4] *Laboratory of Ecohydrology, École Polytechnique Fédérale Lausanne, Lausanne, Switzerland*

S1 File: Supplementary Figures

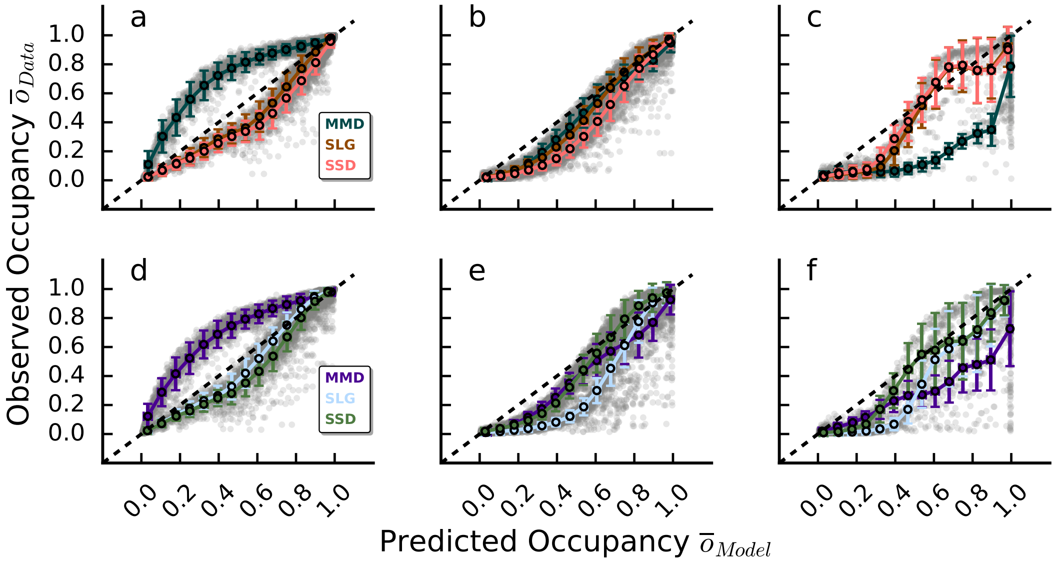

Figure A: The three models capture species occupancy differently. Specifically, the MD model performs poorly at low and high thresholds, while PSLG and MSSD are better at capturing this pattern of diversity. Panels a, b, and c show the comparison between species occupancy prediction and observations for the healthy cohort at low, medium, and high thresholds. Panels d, e, and f show the comparison between prediction and observation for the unhealthy cohort at low, medium, and high cut-off values.

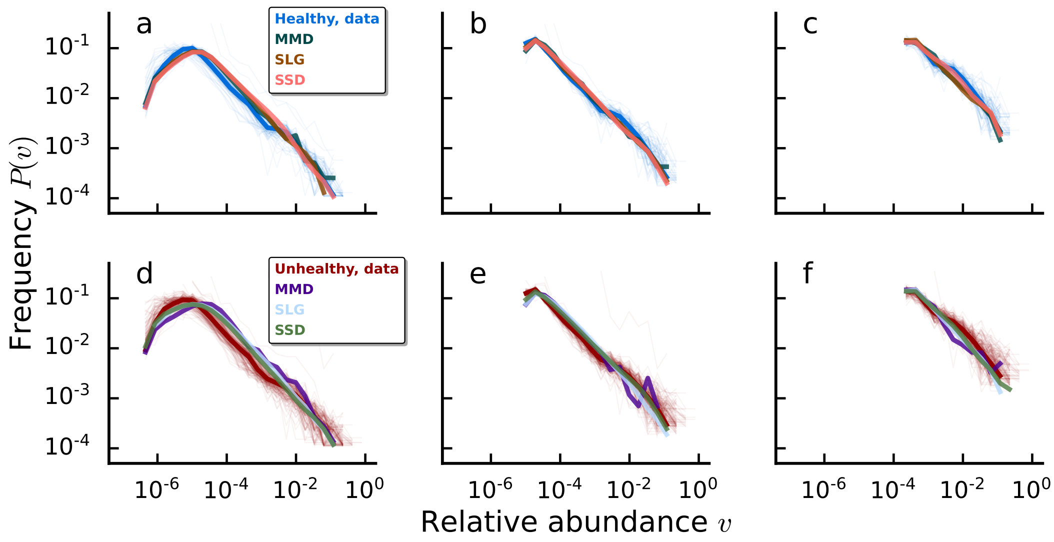

Figure B: All models provide a good description of the species abundance distribution. The thin lines represent the distribution for each sample, while the thick lines represent the mean distribution over all samples for each model. Panels a, b and c show a comparison between the predicted and observed species abundance distribution for the healthy cohort at low, medium, and high thresholds. Panels d, e and f show a comparison between the predicted and observed species abundance distribution for the unhealthy cohort at low, medium, and high cut-off values.

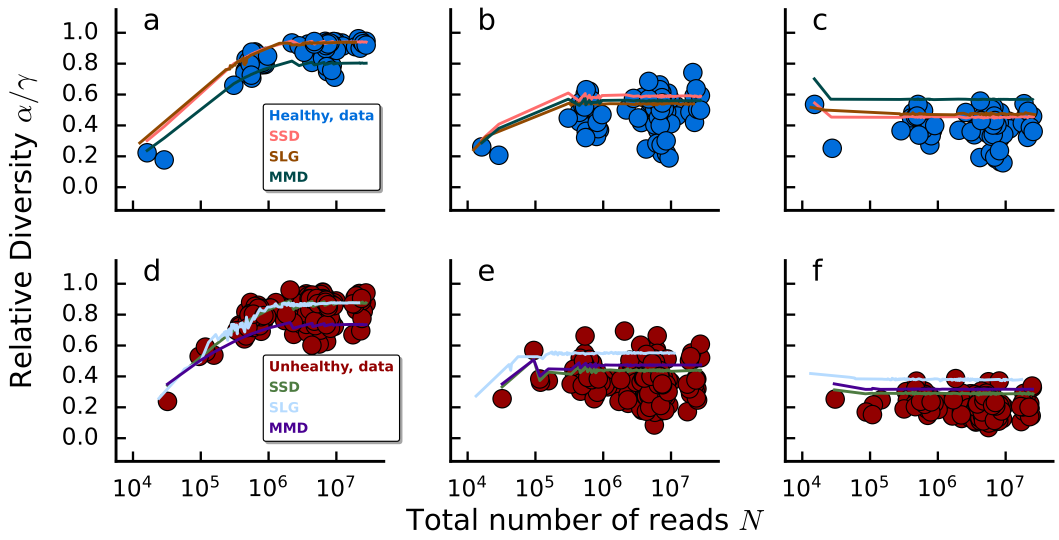

Figure C: Sampling depth-number of species has a non-trivial shape only for low values of the relative abundance threshold. At high values of κ the curve tends to be flattened, a behavior which is qualitatively reproduced by all the models. However, models encoding the $\zeta = 2$ exponent can better capture the average value. Panels a, b, and c show the comparison between species-number of reads relation observed in the data and predicted by the model for the healthy cohort at low, medium, and high thresholds. Panels d, e, and f show the comparison between prediction and observation for the unhealthy cohort at low, medium, and high cut-off values.

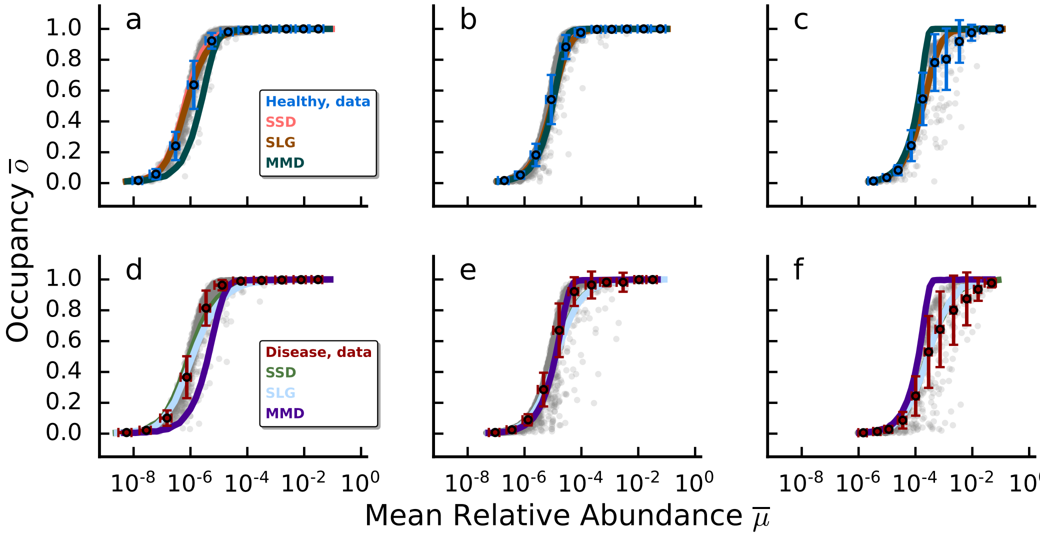

Figure D: Abundance-Occupancy relation is well captured by the PPSLG and MSSD models at all values of κ, while MD fails to describe AO curves except for the case of intermediate cut-off κ. Panels a, b, and c show the comparison between mean relative abundance - occupancy relation observed in the data and predicted by the model for the healthy cohort at low, medium, and high thresholds. Panels d, e, and f show the comparison between prediction and observation for the unuhealthy cohort at low, medium, and high cut-off values.

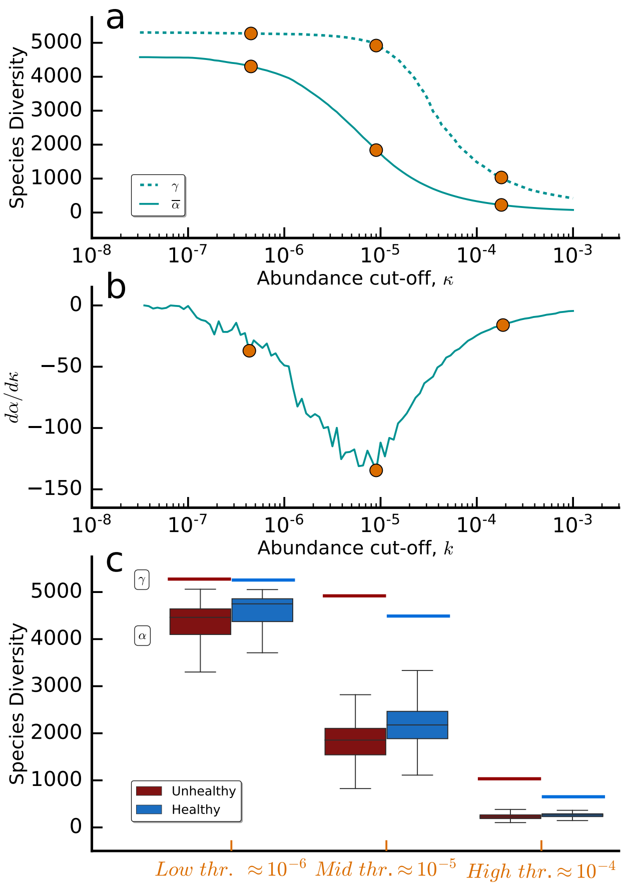

Figure E: Panel a: Depending on the relative abundance cut-off, three regimes can be clearly identified. Starting from low relative abundance cut-off (left side of panel a), the microbiome data have both high α and γ diversity, and their values are comparable. At the intermediate cutoff, sparsity (i.e., the number of absent species) appears, and the gap between α and γ is maximised. Finally, for high thresholds, we have a low-diversity regime in which α and γ are of the same order. are of the same order, but the diversity is low. Panel b: In order to obtain the numerical values of the threshold κ in the main text, we first identified where the application of the threshold led to the maximum decrease in the mean α diversity, obtaining κ_{mid}. The other two values were set as $\kappa_{low} = \kappa_{mid}/20$ and $\kappa_{high} = \kappa_{mid}/20$. The three values are highlighted in orange in the three panels. In panel c, we highlight the α and γ diversity of our data set, distinguishing between healthy and diseased individuals. The box plots are the same (for the data) as those reported in Figure 2 of the main text.

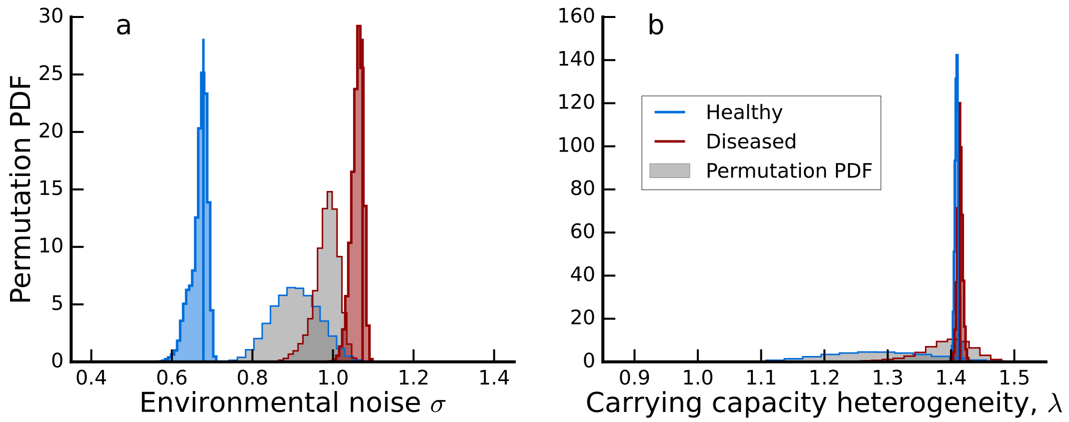

Figure F: Panel a: bootstrap ($n_{Bootstrap\ samples} = 5 \times 10^4$) histogram for environmental noise parameter σ. The grey histogram refers to the permutation test ($n_{permutations} = 5 \times 10^4$) distribution. Solid lines refer to the median of the distribution. Panel b: Same tests and colour code as panel a for the carrying capacity's fluctuations.

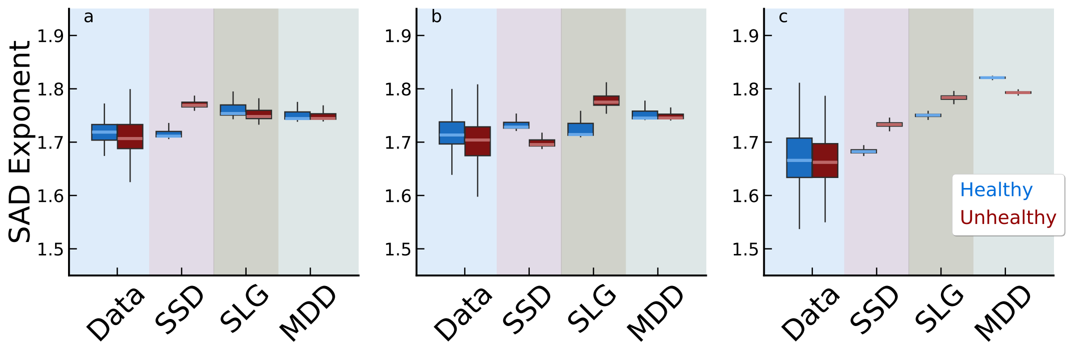

Figure G: SAD right tail exponents are between 1.5 and 1.8. Panel a, b and c show, respectively, the exponents inferred from species abundance distributions obtained for the three different cutoffs κ_{low}, κ_{mid}, κ_{high}

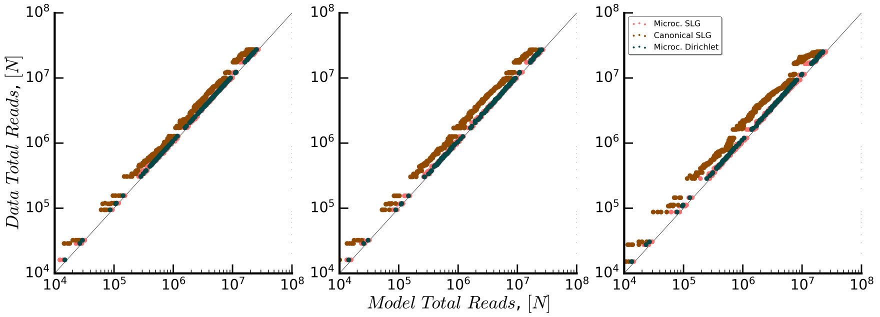

Figure H: The PSLG model systematically underestimates the total number of reads due to Poisson-like sampling, which only preserves the average species abundance. Panels a, b and c show, respectively, the comparison between the number of reads obtained for each model and the data at the three different cutoffs κ_{low}, κ_{mid}, κ_{high}
