# Supplementary material for: Emergent ecological patterns and modelling of gut microbiomes in health and in disease
Source: PLoS Comput Biol. 2024 Sep 27;20(9):e1012482. doi: 10.1371/journal.pcbi.1012482 (PMC11493414; doi:10.1371/journal.pcbi.1012482)
Supplement: S2 File — (PDF) [file pcbi.1012482.s004.pdf]

Emergent Ecological Patterns and Modelling of Gut Microbiomes in Health and in Disease: S2 file

J. Pasqualini[1,*], S. Facchin[2], A. Rinaldo[3,4], A. Maritan[1], E. Savarino[2], S. Suweis[1,*]
[1] *Dipartimento di Fisica "G. Galilei" e INFN sezione di Padova, University of Padova, Padova, Italy*
[2] *Dipartimento di Scienze Chirurgiche, Oncologiche e Gastroenterologiche (DiSCOG), University of Padova, Padova, Italy*
[3] *Dipartimento di Ingegneria Civile, Edile e Ambientale (ICEA), University of Padova, Padova, Italy*
[4] *Laboratory of Ecohydrology, École Polytechnique Fédérale Lausanne, Lausanne, Switzerland*

S2 File: Stratification Analysis

In order to assess the robustness of our results, which were obtained by considering all diseased microbioome samples as equivalent, we performed the main analysis presented in the main text by considering the three different pathological conditions we considered in our analysis, namely Crohn's disease, ulcerative colitis and inflammatory bowel syndrome. First, we checked that the α/γ diversity treadeoff holds when comparing each disease with the control/healthy group: $\alpha_H > \alpha_U$, $\gamma_H < \gamma_U$ where $U \in \{UC,\ CD,\ IBS\}$.

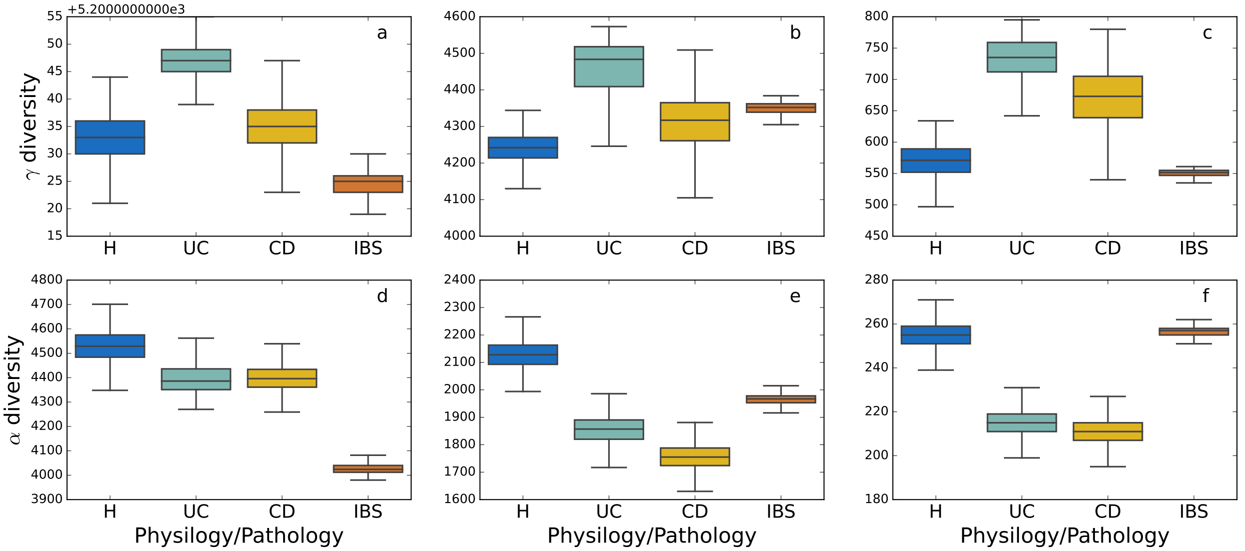

Figure A: Panel a,b,c: γ diversity analysis for different physiological/pathological conditions. Panels a,b,c correspond to data with a low/medium/high relative abundance threshold applied. Panels a,b,c: α diversity analysis for different physiological/pathological conditions. Panel d,e,f correspond to data with low/medium/high relative abundance threshold applied.

As shown in A, this trade-off holds when comparing healthy microbiomes with those affected by Crohn's disease and ulcerative colitis, while it is less clear when comparing inflammatory bowel syndrome microbiomes with control microbiomes. Qualitatively, the result is the same for all thresholds considered. The second aim of our stratification analysis was to check whether the distinction between healthy and unhealthy microbiomes was evident at the individual disease level when looking at the ecological parameters. Similarly to what we have done both in the previous text and in S1 figure F, we estimated the environmental noise σ and the carrying capacity fluctuations λ using a bootstrap procedure. With an uneven number of samples $R_H = 91$, $R_{UC} = 63$, $R_{CD} = 88$, $R_{IBS} = 51$, we took $0.9 \times R_{IBS}$ from each group and considered $n_{\text{Pemutation}} = 10^3$ permutations of samples. In this way we obtained the bootstrap

estimates for σ, λ for the four physiopathological conditions. The values obtained confirm the interpretation presented in the discussion of the main text. For Crohn's disease and ulcerative cholitis, the system is poised on the critical value of the environmental noise level, and species experience a high turnover due to noise-induced extinctions B. Finally, thanks to the bootstrap procedure, for these two cohorts we cannot reject the possibility that such a parameter is above one C.

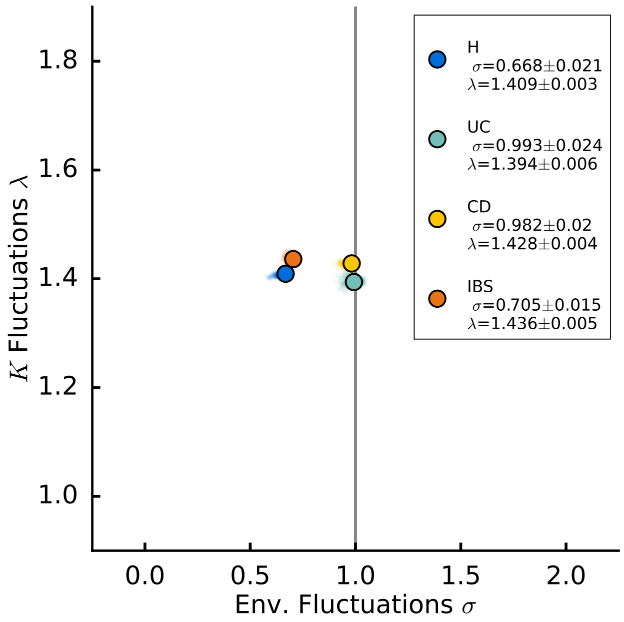

Figure B: According to the physio-pathological condition, healthy and diseased microbiomes arrange themselves in a different regime of the model. Light coloured dots are relative to a single realisation of the parameter fit generated by the bootstrap procedure.

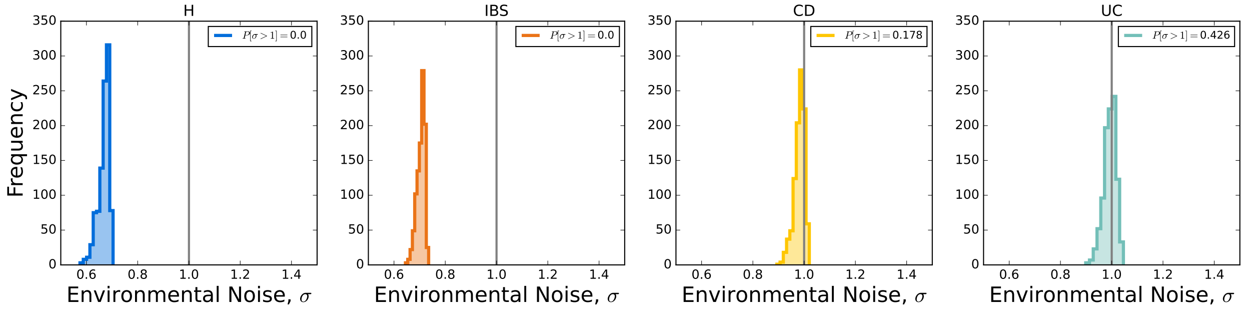

Figure C: Bootstrap histogram for the environmental noise parameter σ for each physiopathological condition.
