# Supplementary material for: Emergent ecological patterns and modelling of gut microbiomes in health and in disease
Source: PLoS Comput Biol. 2024 Sep 27;20(9):e1012482. doi: 10.1371/journal.pcbi.1012482 (PMC11493414; doi:10.1371/journal.pcbi.1012482)
Supplement: S1 Text — (PDF) [file pcbi.1012482.s005.pdf]

Emergent Ecological Patterns and Modelling of Gut Microbiomes in Health and in Disease: S1 Text

J. Pasqualini[1,*], **S. Facchin**[2], **A. Rinaldo**[3,4], **A. Maritan**[1], **E. Savarino**[2], **S. Suweis**[1,*]
[1] *Dipartimento di Fisica "G. Galilei" e INFN sezione di Padova, University of Padova, Padova, Italy*
[2] *Dipartimento di Scienze Chirurgiche, Oncologiche e Gastroenterologiche (DiSCOG), University of Padova, Padova, Italy*
[3] *Dipartimento di Ingegneria Civile, Edile e Ambientale (ICEA), University of Padova, Padova, Italy*
[4] *Laboratory of Ecohydrology, École Polytechnique Fédérale Lausanne, Lausanne, Switzerland*

S1 Text: Model testing

To test how well our models described the data, we introduced goodness-of-fit measures based on the classical R^2 score, as discussed in the main text. We started by noting that the classical score is defined as follows:

$$R^2 = 1 - \frac{\sum_{i=1}^{R}(y_i - f_i)^2}{\sum_{i=1}^{S}(y_i - \overline{y})^2} \tag{1}$$

Where y is an observable associated with a given species. We then adapted the definition, to two different cases we wanted to analyse. Typically considered patterns are of two types: neutral-like patterns and species-species (or sample-sample) patterns. The former can be thought of as a pattern where the only property that distinguish species is stochasticity, such as the abundance-occurrence curve, where the independent variable is the species mean abundance and the dependent variable is the occurrence. Differently, in the latter case, we want to compare the occurrence of a given species in the data with the occurrence produced by our model and here we need to rely on the species identity to make the comparison.

For the AO-relation, we binned the relative abundances of species. For each bin, we obtained the occupancy by averaging the occupancies of all species falling in a given bin. This gave us a curve of the type $\overline{o}_b = \overline{o}(\mu_b)$ for the dataset and each instance of the model. The R^2 score for this model was calculated as follows:

$$R^2_{AO} = 1 - \frac{\sum_{b\in bins}(\overline{o}^{Data}(\mu_b) - \overline{o}^{Model}(\mu_b))^2}{\sum_{b\in bins}(\overline{o}^{Data}(\mu_b) - \langle\overline{o}^{Data}\rangle_{\mu_b})^2} \tag{2}$$

For sake of completeness, we report the R^2_{SSR} formula that we used for the sampling-species curve (which we normalised to the total diversity of each group γ_{group}):

$$R^2_{SSR} = 1 - \frac{\sum_{j=1}^{R}(\delta_j^{Data} - \delta_j^{Model})^2}{\sum_{j=1}^{R}(\delta_j^{Data} - \langle(\delta_j^{Data}\rangle)^2} \tag{3}$$

The above R^2 evaluates how well a given model reproduces the number of observed species in a given sample, given the number of classified reads

As an example of a non-neutral-like pattern, we consider the observed occupancy of species and that predicted by the model. For the occupancy, the R^2 can be written as:

$$R^2_{Occupancy} = 1 - \frac{\sum_{i=1}^{S}(\overline{o}_i^{Data} - \overline{o}_i^{Model})^2}{\sum_{i=1}^{S}(\overline{o}_i^{Data} - \langle\overline{o}^{Data}\rangle)^2} \tag{4}$$

As seen in the *main text*, such patterns are typically harder to capture, as both models we used encode some form of neutrality. Therefore, to compare the data with species we generated a list of species ranked by their mean relative abundance in the empirical communities. Each time we sampled an instance (a table) from our model, we sampled a realisation of the *MAD* which is a set of $S = \gamma$ numbers. Ranking the species according by their relative abundance allowed us to sort each instance of the model in a way so that sampled and real communities were comparable.

The single R^2 score was calculated by comparing the dataset with a random instance of the model. Thus, the final R^2-score we report in the *main text* is the average over 500 instances of the model.
