# Supplementary material for: Emergent ecological patterns and modelling of gut microbiomes in health and in disease
Source: PLoS Comput Biol. 2024 Sep 27;20(9):e1012482. doi: 10.1371/journal.pcbi.1012482 (PMC11493414; doi:10.1371/journal.pcbi.1012482)
Supplement: S2 Text — (PDF) [file pcbi.1012482.s006.pdf]

EMERGENT ECOLOGICAL PATTERNS AND MODELLING OF GUT MICROBIOMES IN HEALTH AND IN DISEASE: S2 TEXT

J. Pasqualini[1,*], S. Facchin[2], A. Rinaldo[3,4], A. Maritan[1], E. Savarino[2], S. Suweis[1,*]
[1] *Dipartimento di Fisica "G. Galilei" e INFN sezione di Padova, University of Padova, Padova, Italy*
[2] *Dipartimento di Scienze Chirurgiche, Oncologiche e Gastroenterologiche (DiSCOG), University of Padova, Padova, Italy*
[3] *Dipartimento di Ingegneria Civile, Edile e Ambientale (ICEA), University of Padova, Padova, Italy*
[4] *Laboratory of Ecohydrology, École Polytechnique Fédérale Lausanne, Lausanne, Switzerland*

S2 Text: Supplementary Methods

S2.1 Statistical Methods

In this section, we briefly introduce the quantities we employed in order to evaluate model performance.

S2.1.1 Bayesian Information Criterion

Bayesian Information Criterion is a quantity employed in the context of Bayesian model selection. It is defined as follows

$$BIC_{Model}(k, \hat{\theta}, R, \overline{x}) = k \ln R - 2 \ln \mathcal{L}_{Model}(\overline{x}|\hat{\theta})$$

Intuitively, it is introduced as a measure of the goodness of a statistical model (characterised by its maximum likelihood model and number of parameters k) for dataset with a given number of samples R. To infer which of the two MADs better describes the empirical mean abundance distribution, we calclated the ratio between the BIC

$$BIC_{ratio} = \frac{BIC_{Log-Normal}(k, \hat{\theta}, R, \overline{x}, \kappa)}{BIC_{Log-Laplace}(k, \hat{\theta}, R, \overline{x}, \kappa)} \tag{1}$$

To test the performance of each model for different values of the species abundance threshold, we introduced the dependence of the relative abundance cut-off κ. In the spirit of the maximum likelihood principle, when comparing two models, with the same number of parameters, this reduces to the comparison of likelihoods, with a minus sign. Thus the model with the lowest BIC should be preferred. In this sense, $BIC_{ratio} > 1$ the Log-Laplace model should be preferred over the Log-Normal model.

S2.1.2 Fitting Taylor's Law

Taylor's Law describes the scaling relation between the mean and the variance of a set of degrees of freedom. In our case, it describes the scaling relation between the average abundance and the variance of the S species present in the system. For absolute abundance, it can be written as

$$\sigma^2_{x_i} = A\overline{x}_i^{\zeta} \tag{2}$$

Importantly, this relation can be obtained when considering relative abundances ($x_i = Xv_i$) as well. In fact, plugging the latter relation into the equation 2 we find that $X^2\sigma^2_{v_i} = X^{\zeta}A\overline{x}_i^{\zeta}$. We note that the "total abundance" terms cancel out only in the case of $\zeta = 2$. Otherwise, the correction $X^{\zeta-2}$ should be taken into account to properly infer A from the data.

This basic theoretical argument can be easily checked by simulating our model with parameters similar to those of the data. In panel a of figure A, we show data are simulated from the *symmetric shifted Dirichlet* model, with parameters

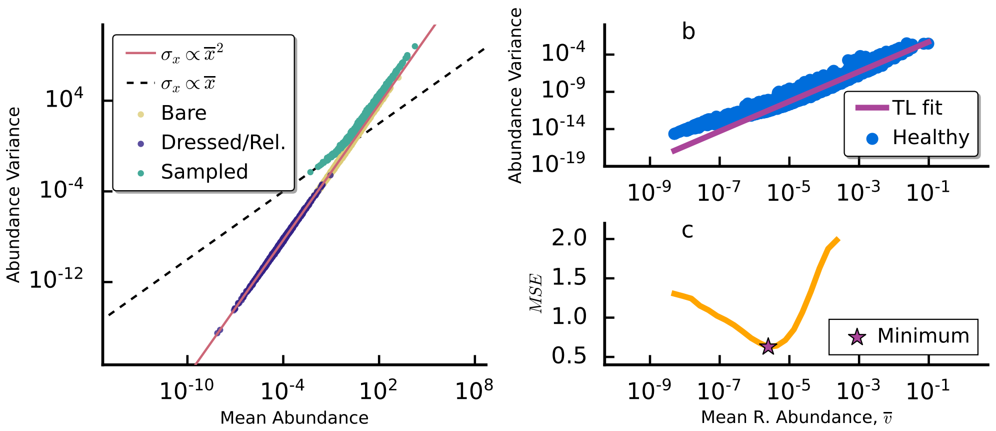

Figure A: Panel a: the shape of TL is not affected by compositionality or sampling. Panel b: we show the data for healthy individuals and the line resulting from our fitting procedure. Panel c: MSE has a minimum when the TL regime starts to dominate over the noise.

compatible with thoose the data ($\gamma = 4500$, $R = 200$, $A = 0.5$, $\lambda = 1.4$), and the number of reads is the same as in the empirical dataset for healthy individuals. Importantly,the Taylor's curve for the sampled data (light green dots in figure A, panel a) exhibits the same transition from Poisson to Taylor scalings. Overall, this makes the inference of the environmental noise $\sigma^{H,U}$ robust to compositionality and sampling. Assuming that the data come from a symmetric scaled Dirichlet distribution with multinomial sampling, we checked that Taylor's law takes the same form in the case of absolute and relative abundances, i.e. considering the former or the latter is equivalent to moving all the points along the same line. In the case of sampled abundances, if the sampling is not sufficiently large, in the rare species regime the sampled abundance is dominated by sampling and Taylor's curve takes a Poisson-like scaling. In order not to let this regime affect our estimation of the parameters of the Taylor law, we proceed as follows. We start by retaining all species and perform a linear fit in log-log scale with a line for which we have fixed the exponent of $\zeta = 2$, and measure the mean squared error (MSE) score. We track how MSE changes by eliminating rare species below a given value $\overline{v}^*$, obtaining a curve of $MSE(\overline{v}^*)$ that saturates where the Taylor regime dominates over Poisson noise. Once this curve reaches a minimum, we stop our procedure and get an estimate of the amplitude of the Taylor law A.

To fit the Multinomial Dirichlet model, which corresponds to $\zeta = 1$, we proceed in the same way, with the only difference that we restrict ourselves a linear model to describe the data $\sigma_v^2 = A_{\text{emp.}}\overline{x}$. As shown above 2 in this case the proportionality constant of the model encodes both the actual Taylor's law proportionality constant A and the community size X and can not be disentangled with the employed fitting procedure.

S2.1.3 Fitting SAD exponents

We obtained the SAD exponent as follows. For each sample, we obtain the species abundance distribution SAD, by making the histogram of the relative abundances. Secondly, we keep values beyond the mode of the distribution, in order to focus on the tails. We then estimate the values of the SAD exponent using *powerlaw* package [1]. For each of the three relative abundance cut-off values κ, we repeat the same procedure for the data and the model. The results are shown in figure G in S1 File.

S2.1.4 Statistical significance of ecological parameters

We wanted to assess the statistical significance of the ecological parameters ($\lambda^{H,U}$ and $\sigma^{H,U}$) we found. To do this, we ran a series of permutation-like tests. The hypothesis we tested, was for each parameter and for each group. We performed a permutation test ($n_{\text{Permutations}} = 5 \times 10^4$) to asses whether the inferred parameters were robust to the permutation of the physiological label. For example, we wanted to test if σ^H is robust to the permutation. We have randomly drawn ($R_H = 91$) samples from the available $R = R_H + R_U$, $n_{\text{Permutations}}$ times and estimated σ, as explained above S2.1.2 and finally performed the permutation test.

For the environmental noise parameter σ, we obtain $p_{H,U} < 5 \times 10^4$, thus rejecting the hypothesis that these values are obtained by chance. For the carrying capacity fluctuation parameter λ we obtain $p_H = 0.93$ and $p_U = 0.69$: thus there is no statistical significance in the value of λ for discriminating the physiological state of the microbiome.

To evaluate the $\lambda^{H,U}$ and $\sigma^{H,U}$ expectation value and uncertainty reported in the main text, we performed a bootstrap procedure ($n_{Bootstrap} = 5 \times 10^4$) retaining a portion ($p = 90\%$) of the samples of each group. We also tested whether the value obtained in the diseased case was statistically significant. Using the bootstrap procedure, we estimate the $p_{Permutation(\sigma^U>1)} = 3.8 \times 10^{-4}$, making the qualitative difference between healthy and diseased AFD significant.

S2.2 Theoretical Methods

S2.2.1 Stochastic Logistic Model

The main assumptions underlying the model proposed by Grilli [2] are that species are non-interacting and that the only source of stochasticity comes from the environment. In the following, the population dynamics model we will use is the *Stochastic Logistic Growth Model*, which is a logistic model with environmental noise. The simplest interpretation of such noise is that it can be obtained as a random perturbation of the growth rate of a species, thus capturing a variety of effects, such as nutrient availability and environmental harshness, and ultimately competition with other species. It can be shown [3] that this model can emerge as a simplification of a weakly interacting *Lotka-Volterra model* with coloured noise. Finally, the stochastic logistic growth model describing the dynamics of species abundance reads [2]:

$$\frac{dx_i}{dt} = \frac{x_i}{\tau_i}(1 - \frac{x_i}{K_i}) + \sqrt{\frac{\sigma_i}{\tau_i}} x_i \xi_i \tag{3}$$

Where K_i is the carrying capacity, τ_i is the relaxation time to the stationary state and σ_i is the width of environmental noise. Mapping this problem onto the corresponding *Fokker-Planck* equation allows us to obtain the stationary distribution for species abundance, a *Gamma distribution*, i.e.,

$$p(x_i|\alpha_i, \beta_i) = \frac{\beta_i^{\alpha_i}}{\Gamma(\alpha_i)} x_i^{\alpha_i - 1} e^{-\beta_i x_i} \tag{4}$$

which can be parametrised in three equivalent ways, each one with a particular scope

$$\begin{aligned} \alpha_i &= \frac{\langle x_i \rangle^2}{\sigma_x^2} = \frac{2}{\sigma_i} - 1 \\ \underbrace{\beta_i}_{\text{Parameters}} &= \underbrace{\frac{\langle x_i \rangle}{\sigma_x^2}}_{\text{Observables}} = \underbrace{\frac{2}{\sigma_i K_i}}_{\text{Ecology}} . \end{aligned} \tag{5}$$

We can easily relate the standard parametrisation of the Γ distribution to its moments and to ecological quantities that appear in the stochastic logistic growth equation. The calculations are simple, and the interested reader can find them in [2]. This model describes the absolute abundances of species, since the values are bounded on the positive real line. They are all independent, so we can obtain the *joint probability density function* (joint pdf) by multiplying the individual species distributions.

Furthermore, combining equations (2) and (5), we can study the two limiting cases of Taylor's law (respectively $\zeta = 1$, $\zeta = 2$), and how such cases impact the moments of the corresponding Gamma distribution. Now, if we insert the ecological parametrization of the moments in the expression for Taylor's Law, we find:

$$\begin{aligned} K_i^2 \frac{\sigma_i}{2}(1 - \frac{\sigma_i}{2}) &= A K_i^{\zeta} (1 - \frac{\sigma_i}{2})^{\zeta} , \\ A &= \frac{\sigma_i K_i}{2} , \quad \text{for } \zeta = 1 , \\ A &= \frac{\sigma}{2 - \sigma} , \quad \text{for } \zeta = 2 . \end{aligned} \tag{6}$$

which highlights the importance of the exponent ζ for the ecological interpretation of the data. In the first case ($\zeta = 1$) we find that the environmental noise is inversely proportional to the carrying capacity. As such it woul follow a log-Normal / log-Laplace distribution. In the second case ($\zeta = 2$), we find that environmental fluctuations strength is the same for all species ($A = \frac{\sigma}{2-\sigma}$), being independent on the carrying capacity of the species. This result tells us that, regardless of how the species adapt to the environment, all of them will face the same fluctuations on the growth rates. From a data analysis point of viwe, as confirmed in figure A, the scaling between mean and variance for relative abundances is not affected by the community size, allowing us to infer in a reliable way σ from the data trough A.

S2.2.2 Derivation of the Scaled Dirichlet Distribution

In order to write a joint pdf that is compatible with the experimental constraints, we need to take compositionality into account. To enforce this constraint, we need to transform the vector of species abundances into a vector on the simplex Δ^S. To achieve this, we need to sample from the original joint pdf only those configurations compatible with a given total population $\sum_{i=1}^S x_i = X$. In the language of statistical mechanics, this implements a (*microcanonical*) constraint that introduces an effective coupling between species:

$$P(\vec{x}|\vec{\alpha},\vec{\beta}) = \frac{1}{Z(\vec{\alpha},\vec{\beta})}[\prod_{i=1}^{S} p(x_i|\alpha_i,\beta_i)]\delta(\sum_{i=1}^{S} x_i - X) \tag{7}$$

The problem can be solved by a change of coordinates $v_i = x_i/X$ and allows us to find the *dressed* joint probability distribution for relative abundances, which was found in another context by [4]. From this dressed probability distribution for abundances, we obtain a family of joint probability distributions, whose configurations are point on Δ^S simplex, i.e.,

$$P(\vec{v}|\vec{\alpha},\vec{\beta}) = \frac{1}{Z(\vec{\alpha},\vec{\beta})}\frac{\prod_{i=1}^{S} v_i^{\alpha_i-1}}{(\sum_{i=1}^{S}\beta_i v_i)^{\alpha_0}} \tag{8}$$

Employing basic properties of Dirac's delta, the derivation is straightforward:

$$\begin{aligned} P(\vec{v}|\vec{\alpha},\vec{\beta}) &= \int d^S x \prod_{i=1}^{S}\left(P_i(x_i)\delta(v_i - \frac{x_i}{\sum_j x_j})\right) = \\ &= \int_0^\infty dX \int d^S x \prod_{i=1}^{S}\left(P_i(x_i)\delta(v_i - \frac{x_i}{X})\right)\delta(\sum_j x_j - X) = \delta(1-\sum_i v_i)\int_0^\infty dX X^{S-1}\prod_i P_i(v_i X) = \\ &= \mathcal{N}\delta(1-\sum_i v_i)\prod_i v_i^{\alpha_i-1}\int_0^\infty dX X^{\sum_i \alpha_i - 1} e^{-X\sum_i \beta_i v_i} = \frac{1}{Z(\vec{\alpha},\vec{\beta})}\frac{\prod_i v_i^{\alpha_i-1}}{(\sum_i \beta_i v_i)^{\sum_i \alpha_i}}\delta(1-\sum_i v_i), \end{aligned} \tag{9}$$

Where $\mathcal{N} = \prod_i \beta_i^{\alpha_i}/\Gamma(\alpha_i)$ is constant and $Z(\vec{\alpha},\vec{\beta}) = \prod_{i=1}^S \beta_i^{\alpha_i}/B(\vec{\alpha})$ is the normalisation constant of the scaled Dirichlet distribution family.

Following on the work of Monti et al [4] the former distribution can be obtained in a more heuristic way. Starting with a Dirichlet distribution $\vec{y} \sim D(\vec{y}|\vec{\alpha})$ and introducing the following change of coordinates $v_i = \beta_i y_i/(\sum_{j=1}^S \beta_j y_j)$ which encodes the *rescaling* of all the variables and then requiring that the sum-to-one constraint still holds, one obtains the we use in the main text. Due to the rescaling nature of the former coordinate transformation, the resulting distribution is called *Scaled Dirichlet Distribution*.

S2.2.3 Fitting the mean abundance distribution from the data

Here, we show the effect of compositionality on the mean abundance distribution. The key idea is to evaluate the probability density of the normalized averages, which is the only measurable quantity from the data. The result is that, in the limit of a large number of species (which is our case) the location parameter μ is washed out by compositionality, while the fluctuation parameter λ is slightly modified with an additive contribution. However in the limit of a large number of species, such contribution is negligible, and we can avoid to consider it. Finally, the fitted λ from compositional data can be confused with the original one. Now, let's consider S log-normally distributed random variables for the absolute abundances (we will use the exponential representation in terms of gaussian variables $e^{\mu_A+\lambda_A z}$) and define the following new set of random compositinal variables $x_{i=1,\dots,S}$:

$$x_i = \frac{e^{\mu_A+\lambda_A z_i}}{\sum_{i=1}^{S} e^{\mu_A+\lambda_A z_i'}} = \frac{\cancel{e^{\mu_A}} e^{\lambda_A z_i}}{\cancel{e^{\mu_A}}\sum_{i=1}^{S_{\text{tot}}} e^{\lambda_A z_i'}} \tag{10}$$

so that $\sum_i x_i = 1$. Writing down the compositional averages is enough to show that the "localization" for the absolute abundances μ_A is washed out by compositionality. Now, if we achieve to write the denominator as another log-normal variable, we can evalluate x_i as the ratio of two log-normal variables, which is easy to finally compute.

$$x_i = \frac{e^{\lambda_A z_i}}{\sum_{i=1}^{S_{\text{tot}}} e^{\lambda_A z_i'}} = \frac{e^{\lambda_A z}}{e^{\rho+\lambda_{\text{corr.}} z'}} = e^{-\rho+\sqrt{\lambda_A^2+\lambda_{\text{corr.}}^2} z''} \tag{11}$$

where z and z'' are standard gaussian variables. We can find the parameters ρ and $\lambda^2_{\text{corr.}}$ by matching the moments of our ansatz with its the original expression. We find:

$$\begin{aligned} \langle \sum_{i=1}^{S} e^{\lambda_A z_i'} \rangle &= S e^{\frac{\lambda_A^2}{2}} = e^{\rho + \frac{\lambda^2_{\text{corr.}}}{2}} \\ \text{var}(\sum_{i=1}^{S} e^{\lambda_A z_i'}) &= S^2 (e^{2\lambda_A^2} - e^{\lambda_A^2}) = (e^{\lambda_{\text{corr.}}} - 1) e^{2\rho + \lambda^2_{\text{corr.}}} \end{aligned} \tag{12}$$

Which solutions are:

$$\begin{aligned} \lambda^2_{\text{corr.}} &= \ln\left(\frac{e^{\lambda_A^2} - 1}{S} + 1\right) \\ \rho &= \ln S + \frac{\lambda_A^2}{2} - \frac{\lambda^2_{\text{corr.}}}{2} \end{aligned} \tag{13}$$

Finally, the expression for λ reads:

$$\lambda^2 = \lambda_A^2 + \ln\left(\frac{e^{\lambda_A^2} - 1}{S} + 1\right) \tag{14}$$

Assuming $S \gg e^{\lambda^2} - 1$, we can use the former reuslt to write an asymptotic expansion for λ, which reads:

$$\lambda = \lambda_A \sqrt{1 + \frac{e^{\lambda_A^2} - 1}{\lambda_A^2 S}} \approx \lambda_A (1 + \frac{e^{\lambda_A^2} - 1}{2\lambda_A^2 S} + \mathcal{O}(S^{-2})) \approx \lambda_A + \mathcal{O}(S^{-1}) \tag{15}$$

Using the values for the scale parameter and number of species similar to the one fouonnd in the data, namely $\sigma_A^2 \approx 1.4$ and $S \approx 4.5 \times 10^3$, the correction can be considered negligible, being $\lambda^2_{\text{corr.}} \approx 1.3 \times 10^{-3}$. This simple calculation suggest us that $\lambda \approx \lambda_A$ and confirms that we can not access μ_A, as long as we deal with compositional data. Considering the former results, we can safely assume $\lambda = \lambda_A$. Importantly, we fitted λ from the data with no cut-off. This ensured the possibility to generate synthetic data in a coherent way with the empirical ones.

S2.2.4 Invariance of SSD from the MAD μ parameter

So far, we have obtained the scaled Dirichlet distribution, a flexible statistical model to derive both MD and MSSD, as shown in the main text. From now on, for simplicity we will drop the δ, but remember that, as soon as we consider compositional variables, this constraint always holds. Noting that, thanks to the empirical form of mean abundance distributions, mean abundances can be written in terms of the exponential of a random (e.g. Laplace or Normal) variable of the kind $\overline{x}_i = e^{\mu+\lambda z_i} = \rho e^{\lambda z_i} = \rho \overline{x}_i'$ where we introduced $\rho = e^{\mu}$ and $\overline{x}_i' = e^{\lambda z_i}$. We now introduce an argument to show that, only in the case of the Taylor exponent $\zeta = 2$, the dressed probability distribution of species abundance is insensitive respect to ρ and, hence to from μ. In other terms, the only member of the scaled dirichlet distribution family insensitive to the scale of mean abundance μ is the *Symmetric Scaled Dirichlet* distribution, corresponding to the case $\zeta = 2$. Now, recalling the Taylor Law scaling between species mean and standard deviation

$$\sigma_{x_i}^2 = A \overline{x}_i^{\zeta}, \tag{16}$$

We can see how the scaled dirichlet distribution familiy parameters are affected by ρ:

$$\begin{aligned} \alpha_i &= \frac{\overline{x}_i^{2-\zeta}}{A} = \rho^{2-\zeta}\frac{\overline{x}_i'^{2-\zeta}}{A} = \rho^{2-\zeta}\alpha_i' \\ \beta_i &= \frac{\overline{x_i}^{1-\zeta}}{A} = \rho^{1-\zeta}\frac{\overline{x}_i'^{1-\zeta}}{A} = \rho^{1-\zeta}\beta_i' \end{aligned} \tag{17}$$

To have more concise calculations, we can treat separetly the partition function and the density function, checking that they covary in the same way, namely

$$\begin{aligned} p(\vec{v}|\vec{\alpha},\vec{\beta}) =& \frac{f(\vec{v}|\vec{\alpha},\vec{\beta})}{Z(\vec{\alpha},\vec{\beta})} = \frac{f(\vec{v}|\rho^{2-\zeta}\alpha_i',\rho^{1-\zeta}\beta_i')}{Z(\vec{v}|\rho^{2-\zeta}\alpha_i',\rho^{1-\zeta}\beta_i')} \\ =& \frac{Q(\rho)f(\vec{v}|\alpha_i',\beta_i')}{Q(\rho)Z(\vec{v}|\alpha_i',\beta_i')} = \frac{f(\vec{v}|\vec{\alpha'},\vec{\beta'})}{Z(\vec{\alpha'},\vec{\beta'})} = p(\vec{v}|\vec{\alpha'},\vec{\beta'}) \end{aligned} \tag{18}$$

In our case, this requires that

$$\begin{aligned} f(\vec{v}|\vec{\alpha},\vec{\beta}) &= \frac{\prod v_i^{\alpha_i}}{(\sum_j \beta_j v_j)^{\alpha_0}} = \frac{1}{\rho^{(1-\zeta)\alpha_0\rho^{2-\zeta}}}\frac{\prod v_i^{\rho^{2-\zeta}\alpha_i'}}{(\sum_j \beta_j' v_j)^{\rho^{2-\zeta}\alpha_0'}} = \frac{1}{\rho^{(1-\zeta)\alpha_0\rho^{2-\zeta}}} f(\vec{v}|\rho,\zeta,\vec{\alpha}',\vec{\beta}') \\ Z(\vec{\alpha},\vec{\beta}) &= \frac{B(\vec{\alpha})}{\prod_i \beta_i^{\alpha_i}} = \frac{B(\rho^{2-\alpha}\vec{\alpha'})}{\prod_i \beta_i'^{\alpha_i'}} = \frac{1}{\rho^{(1-\zeta)\alpha_0\rho^{2-\zeta}}} Z(\vec{v}|\rho,\zeta,\vec{\alpha}',\vec{\beta}') \end{aligned} \tag{19}$$

Taking the ratio of the two former expression:

$$\frac{f(\vec{v}|\vec{\alpha},\vec{\beta})}{Z(\vec{\alpha},\vec{\beta})} = \frac{f(\vec{v}|\rho,\zeta,\vec{\alpha}',\vec{\beta}')}{Z(\rho,\zeta,\vec{\alpha}',\vec{\beta}')}, \tag{20}$$

It is trivial to see that it can only be satisfied in the case $\zeta = 2$. We conclude that the value of the Taylor exponent $\zeta = 2$ and the hard compositional constraint (indeed, of the MSSD) are features that allow to avoid fitting the μ parameter appearing in the MAD. In our simulations, we set $\mu = 0$ for the MSSD.

References

[1] Aaron Clauset, Cosma Rohilla Shalizi, and Mark EJ Newman. Power-law distributions in empirical data. *SIAM review*, 51(4):661–703, 2009.

[2] Jacopo Grilli. Macroecological laws describe variation and diversity in microbial communities. *Nature communications*, 11(1):1–11, 2020.

[3] Felix Roy, Giulio Biroli, Guy Bunin, and Chiara Cammarota. Numerical implementation of dynamical mean field theory for disordered systems: Application to the lotka–volterra model of ecosystems. *Journal of Physics A: Mathematical and Theoretical*, 52(48):484001, 2019.

[4] V. Pawlowsky-Glahn G.S. Monti, G. Mateu-Figueras and J.J. Egozcue. The shifted-scaled dirichlet distribution in the simplex. In *Proceedings of the 4th International Workshop on Compositional Data Analysis*, 2011.
