# Supplementary material for: Emergent ecological patterns and modelling of gut microbiomes in health and in disease
Source: PLoS Comput Biol. 2024 Sep 27;20(9):e1012482. doi: 10.1371/journal.pcbi.1012482 (PMC11493414; doi:10.1371/journal.pcbi.1012482)
Supplement: S3 Text — (PDF) [file pcbi.1012482.s007.pdf]

# EMERGENT ECOLOGICAL PATTERNS AND MODELLING OF GUT MICROBIOMES IN HEALTH AND IN DISEASE: S3 TEXT

**J. Pasqualini[1,*], S. Facchin[2], A. Rinaldo[3,4], A. Maritan[1], E. Savarino[2], S. Suweis[1,*]**
[1] *Dipartimento di Fisica "G. Galilei" e INFN sezione di Padova, University of Padova, Padova, Italy*
[2] *Dipartimento di Scienze Chirurgiche, Oncologiche e Gastroenterologiche (DiSCOG), University of Padova, Padova, Italy*
[3] *Dipartimento di Ingegneria Civile, Edile e Ambientale (ICEA), University of Padova, Padova, Italy*
[4] *Laboratory of Ecohydrology, École Polytechnique Fédérale Lausanne, Lausanne, Switzerland*

## S3 Text: Metagenomic Pipeline Implementation

All metagenomic samples were available as **.fastq** files. FASTQ is a de facto standard format for storing the results of high-throughput sequencing experiments. It is a text file containing both sequences and a quality score for each nucleotide obtained, where each letter in the sequence is assigned a probability that the base represented is correct.

The first step was to implement an automatic download tool. It only needed the accession code for the metagenomic sample and provided as output the requested **.fastq** file.

Prior to taxonomic classification, we followed standard pre-processing procedures in metagenomics, which included two steps. The first step of pre-processing was performed with Trimmomatic [1] with the aim of removing both reads too short (here we set a minimum read length of $70pb$) to gain precision in taxonomic classification, or with low average quality. The first was to decontaminate the sample with host genetic material. This step was performed using the Bowtie2 [2] utility, which compares each read in the sample with a reference database of human DNA sequences, and if a read matches a human sequence (from the GRch18 genome), it is removed from the sample.

After pre-processing, the samples were ready for taxonomic profiling using Kaiju [3]. The final output of the classification was **.csv** files reporting the number of reads assigned at the species level, from which we built our abundance vector for each patient. All of the former steps were integrated into a unique pipeline, implemented with the workflow manager SnakeMake [4]. We merged all the abundance vectors and obtained the central object of our quantitative analysis, which we will refer to as taxonomic matrices (TMs). We obtained two tables, one for the healthy and one for the unhealthy groups. TMs are matrices where each row represents a taxa and each column represents a sample, so that each entry represents the number of reads assigned to a bacterial species in a given sample:

$$T_{s,p} = \#\{ \textit{ number of reads assigned to the species } s \textit{ in the patient } p \ \} \tag{1}$$

The taxonomic table associated with the healthy cohort has a number of rows and a number of columns, respectively $S_h = 89$ $P_h = 75$. For the unhealthy table, we have $S_u = 130$ $P_u = 84$, so our dataset is almost balanced.

## References

[1] Anthony M Bolger, Marc Lohse, and Bjoern Usadel. Trimmomatic: a flexible trimmer for illumina sequence data. *Bioinformatics*, 30(15):2114–2120, 2014.

[2] Ben Langmead and Steven L Salzberg. Fast gapped-read alignment with bowtie 2. *Nature methods*, 9(4):357–359, 2012.

[3] Peter Menzel, Kim Lee Ng, and Anders Krogh. Fast and sensitive taxonomic classification for metagenomics with kaiju. *Nature communications*, 7(1):11257, 2016.

[4] Johannes Köster and Sven Rahmann. Snakemake—a scalable bioinformatics workflow engine. *Bioinformatics*, 28(19):2520–2522, 2012.
